# O-GlcNAcylation of PERIOD regulates its interaction with CLOCK and timing of circadian transcriptional repression

**DOI:** 10.1371/journal.pgen.1007953

**Published:** 2019-01-31

**Authors:** Ying H. Li, Xianhui Liu, Jens T. Vanselow, Haiyan Zheng, Andreas Schlosser, Joanna C. Chiu

**Affiliations:** 1 Department of Entomology and Nematology, College of Agricultural and Environmental Sciences, University of California, Davis, Davis, CA, United States of America; 2 Rudolf Virchow Center for Experimental Biomedicine, University of Würzburg, Würzburg, Germany; 3 Biological Mass Spectrometry Facility, Robert Wood Johnson Medical School and Rutgers, the State University of New Jersey, Piscataway, NJ, United States of America; Washington University in Saint Louis School of Medicine, UNITED STATES

## Abstract

Circadian clocks coordinate time-of-day-specific metabolic and physiological processes to maximize organismal performance and fitness. In addition to light and temperature, which are regarded as strong zeitgebers for circadian clock entrainment, metabolic input has now emerged as an important signal for clock entrainment and modulation. Circadian clock proteins have been identified to be substrates of O-GlcNAcylation, a nutrient sensitive post-translational modification (PTM), and the interplay between clock protein O-GlcNAcylation and other PTMs is now recognized as an important mechanism by which metabolic input regulates circadian physiology. To better understand the role of O-GlcNAcylation in modulating clock protein function within the molecular oscillator, we used mass spectrometry proteomics to identify O-GlcNAcylation sites of PERIOD (PER), a repressor of the circadian transcriptome and a critical biochemical timer of the *Drosophila* clock. *In vivo* functional characterization of PER O-GlcNAcylation sites indicates that O-GlcNAcylation at PER(S942) reduces interactions between PER and CLOCK (CLK), the key transcriptional activator of clock-controlled genes. Since we observe a correlation between clock-controlled daytime feeding activity and higher level of PER O-GlcNAcylation, we propose that PER(S942) O-GlcNAcylation during the day functions to prevent premature initiation of circadian repression phase. This is consistent with the period-shortening behavioral phenotype of *per*(S942A) flies. Taken together, our results support that clock-controlled feeding activity provides metabolic signals to reinforce light entrainment to regulate circadian physiology at the post-translational level. The interplay between O-GlcNAcylation and other PTMs to regulate circadian physiology is expected to be complex and extensive, and reach far beyond the molecular oscillator.

## Author summary

Circadian clocks are self-sustained, endogenous pacemakers that enable organisms to anticipate daily environmental changes and resource abundance to perform specific time-of-day activities and achieve optimal survival. Multiple time cues are interpreted by circadian clocks to facilitate synchrony between organisms and their environment. A large body of work have identified light and temperature as important zeitgebers. More recent works highlight the significance of metabolic cues as signals to entrain and modulate circadian clocks to drive proper rhythms of physiology and behavior. Metabolic input, primarily through clock-controlled feeding activity, can regulate circadian physiology through multiple pathways. Some of these pathways are unknown while others, such as the O-GlcNAcylation of clock proteins, are just emerging. In this study, we utilized mass spectrometry proteomics to identify O-GlcNAcylation sites of the *Drosophila* PERIOD (PER) protein, a key regulator of the clock, and performed site-specific functional characterization of PER O-GlcNAcylation. Our results support that PER(Ser942) O-GlcNAcylation, a nutrient-sensitive protein modification that is expected to be more abundant during feeding period, prevents newly synthesized PER from prematurely performing its function during daytime and therefore restricts its activity to nighttime when flies are fasting. This study provides new insights into the mechanisms linking nutrient input and circadian physiology.

## Introduction

Circadian clocks are endogenous protein machines that integrate external time cues and internal metabolic states to impose temporal organization on physiology, metabolism, and behavior (reviewed in [1–2]). They allow organisms from all kingdoms of life, which experience perpetual 24-hour day-night cycles, to anticipate daily environmental changes and execute biological tasks, from molecular to behavioral levels, at the optimal time of day. Over the years, great progress in elucidating the molecular mechanisms driving circadian rhythms has been made by studying the core circadian oscillator. The molecular oscillator consists of two interlocked transcriptional translational feedback loops (TTFLs) that produce daily oscillations in clock mRNAs and proteins to drive rhythms in diverse cellular processes. During the day and into the early parts of the night, two basic helix-loop-helix (bHLH)-PAS transcription factors, CLOCK (CLK) and CYCLE (CYC; homolog of BMAL1 in mammals), activate transcription of their own repressors, *period* (*per*) and *timeless* (*tim*) and other clock-controlled output genes [3]. After a time-delay and TIM-assisted entry into the nucleus [4], PER interacts with and inhibits CLK-CYC activity [5]. This repression is relieved upon proteasomal-dependent degradation of PER during late night into early morning, thus initiating another round of CLK-CYC-mediated transcription [6–7]. Among CLK-activated genes are two bZIP transcription factors, *vrille (vri*) and *par domain protein 1*ε *(Pdp1*ε) [8–9]. Due to differential kinetics of VRI and PDP1ε protein accumulation, VRI accumulates first and inhibits *Clk* expression. As VRI level decreases, PDP1ε accumulates and activates *Clk* transcription, and the cycle of *per/tim* expression starts again. More recent evidence however suggested that the main role of VRILLE could be to control clock output by driving rhythms in expression of the neuropeptide PDF (Pigment Dispersing Factor) and neuronal arborization [10]. CLOCKWORK ORANGE (CWO) is another direct CLK target that feedbacks and represses CLK activity by competing with CLK-CYC complexes for E-box binding at circadian promoters [11]. The TTFLs are synchronized to the 24-hour day-night cycle through light-dependent degradation of TIM [12–14], which interacts with the photoreceptor CRYPTOCHROME 1 (CRY1) [15].

To extend the duration of the TTFL to last a 24-hr circadian cycle, post-translational regulation of core clock proteins overlays on the TTFL and has been recognized to be critical in maintaining the functionality of the circadian oscillator (reviewed in [1, 16]). In *Drosophila*, the phase-specific phosphorylation state of PER is closely linked to its time-of-day specific function and the speed of the oscillator [17–21]. *De novo* synthesized hypophosphorylated PER goes through a multi-site phosphorylation program that progressively increases its phospho-occupancy until it gets hyperphosphorylated. In particular, phosphorylation at a N-terminal phosphodegron targets PER for degradation in a proteasome-dependent manner [17]. In a study by Robles et al. [22], 25% of the 20,000 phosphosites identified in mouse liver proteins were found to oscillate over the circadian cycle. This suggests that widespread and dynamic oscillations in phosphorylation occur beyond core circadian transcription factors to transition cellular proteins between functional states to regulate circadian physiology.

More recently, O-GlcNAcylation has emerged as another PTM that can regulate the temporal function and activity of circadian transcription factors [23–25]. In contrast to protein phosphorylation, which is mediated by a wide selection of kinases and phosphatases, protein O-GlcNAcylation is regulated by a single pair of enzymes with opposing functions [26]. O-GlcNAc transferase (OGT) and O-GlcNAcase (OGA) facilitate the O-GlcNAcylation and de-O-GlcNAcylation of cellular proteins respectively. In *Drosophila*, PER and CLK have been identified as substrates of O-GlcNAcylation, and there is evidence from overexpression of *ogt* to support that O-GlcNAcylation of these clock transcription factors regulates their nuclear translocation, stability, and transcriptional activity [23–24]. In mammalian clocks, BMAL1 and CLOCK have been shown to be rhythmically O-GlcNAcylated over the circadian cycle, and O-GlcNAcylation functions to counteract ubiquitination to stabilize these proteins [25]. In a separate study, PER2 was shown to be modified by O-GlcNAcylation at the S662-S674 region, which is important for regulating clock speed via CK1 phosphorylation [24]. A S662G mutation in humans is known to cause the familial advanced sleep phase syndrome (FASPS) [27]. Interestingly, S662 can also be O-GlcNAcylated, suggesting *in vivo* interplay between phosphorylation and O-GlcNAcylation in this domain. Increasingly, the interplay between phosphorylation and O-GlcNAcylation is shown to be prevalent in the regulation of diverse cellular processes (reviewed in [1]). As O-GlcNAcylation is a nutrient-sensitive PTM that relies on the availability of UDP-GlcNAc, an end product of the hexamine biosynthetic pathway (HBP), it is expected that levels of cellular protein O-GlcNAcylation may be highly dependent on daily feeding-fasting cycles.

Many unknowns regarding PTM regulation of clock proteins remain, including the mechanisms by which phase-specific phosphorylation and O-GlcNAcylation collaborate to regulate their time-of-day functions. A significant barrier to understanding these mechanisms is the identification of O-GlcNAcylated residues. In fact, although both *Drosophila* PER and CLK are known to be O-GlcNAcylated, specific O-GlcNAcylated residues have not been identified [23–24]. The effects of O-GlcNAcylation on these clock proteins have only been investigated by global overexpression and knockdown of *ogt* and *oga*, which impact all O-GlcNAcylated residues simultaneously. Lessons learned from previous studies on clock kinases [17–21] highlight the likelihood that valuable mechanistic insights may be overlooked by global manipulation in PTM enzyme expression. Other important questions concern the temporal requirement of clock protein O-GlcNAcylation and the relationship between protein O-GlcNAcylation status and feeding-fasting cycles.

In this study, we sort to understand how metabolic input influences the O-GlcNAcylation status of PER and regulates its function. We used Mass Spectrometry (MS) proteomics to identify PER O-GlcNAcylation and phosphorylation sites from adult flies, and characterized the function of site-specific O-GlcNAcylation events *in vivo*. We focused on PER as its phase-specific function has been shown to be highly dependent on its progressive phosphorylation program over the circadian cycle. Investigating PER O-GlcNAcylation can therefore set the stage for understanding the interactions between phosphorylation and O-GlcNAcylation in regulating its circadian function. We observe that loss of O-GlcNAcylation at multiple residues affect PER repressor function. In particular, loss of O-GlcNAcylation at PER(S942), which is located in the PER-CLK interaction or CLK binding domain (CBD), leads to stronger PER-CLK interaction and premature entry into the circadian repression phase. Conversely, overexpression of OGT in clock neurons weakens PER-CLK interaction, contributing to its period-lengthening phenotype in locomotor activity rhythms. Finally, we report that daily rhythms of PER O-GlcNAcylation in adult head tissues correlate with feeding-fasting cycles. This correlation is expected to be even stronger in peripheral tissues, which are more sensitive to metabolic signals. Specifically, PER O-GlcNAcylation exhibits circadian rhythmicity and is higher during the day when flies are actively feeding. Our results suggest that metabolic input collaborates with other entrainment signals to regulate time-of-day PER function in circadian transcription.

## Results

### Mass spectrometry analysis identifies PER O-GlcNAcylation and phosphorylation sites in fly tissues

Although O-GlcNAcylation has been shown to influence PER function, specific residues that are modified by O-GlcNAcylation have not been identified [23–24]. This represents a critical barrier to understand the function of site-specific O-GlcNAcylation events and the interplay between O-GlcNAcylation and phosphorylation to regulate the phase-specific functions of PER. We therefore sought to identify PER O-GlcNAcylation sites and obtain temporal data on their occupancy. We purified FLAG-tagged PER from heads of *wper*^*0*^*; p{*3XFLAG-*per*(WT)} flies at specific time-points over the circadian cycle using FLAG affinity purification and performed quantitative mass spectrometry (MS) to obtain a circadian profile of PER phosphorylation and O-GlcNAcylation using fly tissues as starting materials. Although we attempted to perform this study using both fly head and body tissues, we were only able to identify PER O-GlcNAcylation sites in head tissues since we were unable to pull down sufficient PER proteins in bodies for comprehensive PTM identification. Nevertheless, we postulate that PER residues that are O-GlcNAcylated in head tissues will also be O-GlcNAcylated in peripheral tissues due to the ubiquitous expression of *ogt* [28]. To enable quantitation of PTM sites, flies were fed with ^15^N-labeled (heavy) yeast or ^14^N (light) yeast for two generations to ensure complete labeling in flies (herein termed ^15^N and ^14^N flies) [29]. To ensure that ^15^N-fed and ^14^N-fed flies show similar behavioral rhythms, we examined their locomotor activity rhythms using *Drosophila* activity monitoring [30]. Both types of flies displayed strong behavioral rhythms with periods close to 24-hr in constant darkness (S1A and S1B Fig). Furthermore, we measured PER daily abundance in ^15^N and ^14^N flies to confirm that temporal expression of PER was not altered as a result of the diets and observed no difference (S1C Fig). To profile PER PTMs *in vivo*, ^15^N and ^14^N flies were collected at six time-points over the circadian cycle (ZT1, 3, 12, 16, 20, 24), and protein extracts from heads of ^15^N and ^14^N flies were separately subjected to FLAG affinity purification (AP) prior to sample preparation for LC-MS/MS analysis (S2A and S2B Fig). Relative PTM quantification was achieved by pooling purified PER from all ^14^N samples, and aliquoting equal amounts of pooled ^14^N fractions to each of the six ^15^N purified PER samples at a 1:1 ratio prior to MS analysis (S2C Fig).

**Fig 1:**
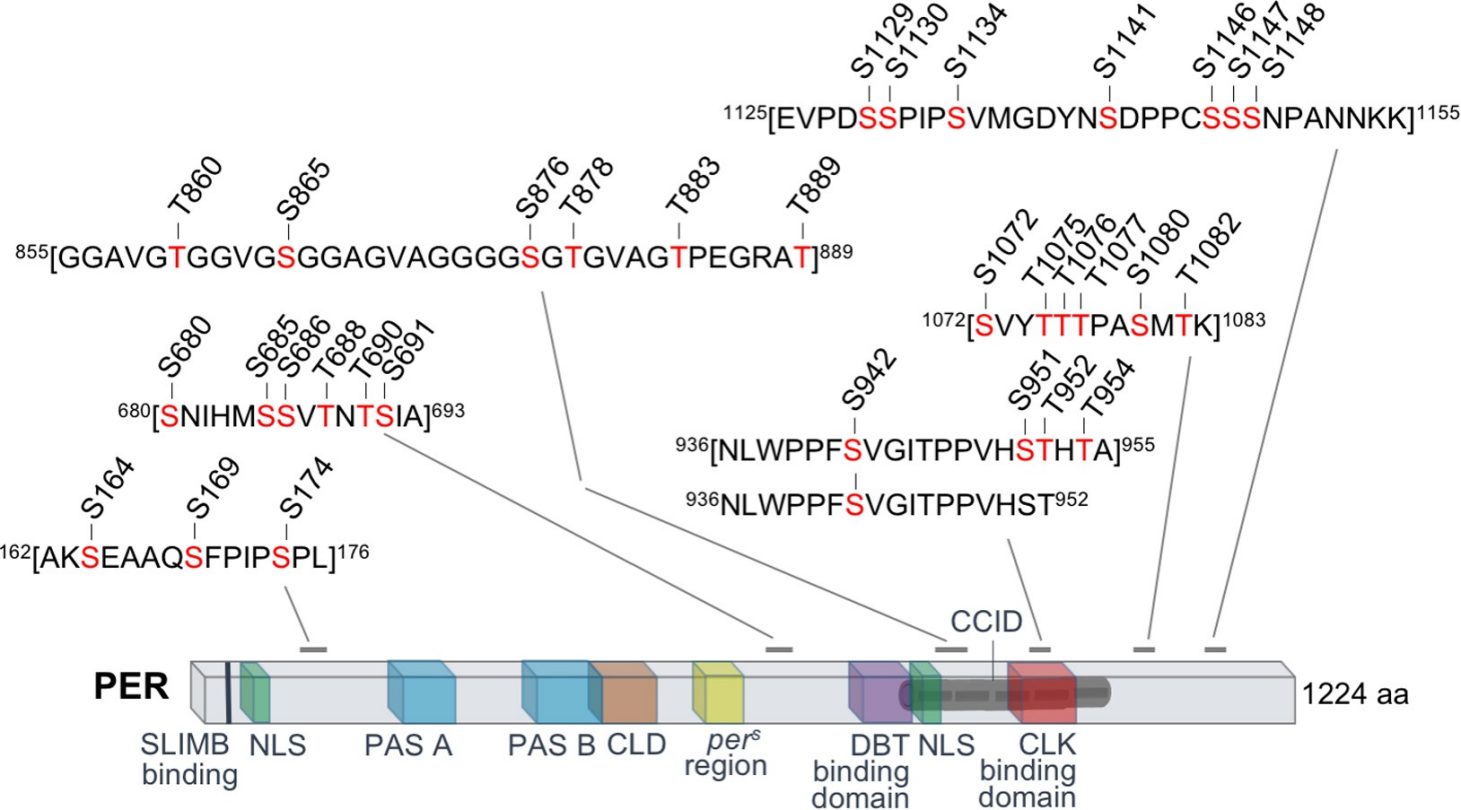
MS proteomic analysis identifies *Drosophila* PER O-GlcNAcylation sites. PER O-GlcNAcylation sites mapped onto PER coding region. Peptide sequences within square brackets include potential O-GlcNAc-modified residues (highlighted in red). Previously characterized functional domains include SLIMB binding site (pS47) [17]; PAS-A (aa240-293) [33, 34]; PAS-B (aa 365–454) [33, 34]; *per*^*s*^ region (aa585-601) [35]; Cytoplasmic Localization Domain (CLD) (aa450-513) [31]; dCLK:CYC Inhibition Domain (CCID) (aa764-1034) [31]; Nuclear Localization Sequence (NLS) (aa814-840) [31]; DBT Binding Domain (DBT BD) (aa755-809) [36]; CLK Binding Domain (CBD) (aa926-977) [32]. Corresponding Mascot scores of modified peptides are presented in Table 1.

Despite the large amount of fly head tissues we used for our protein extraction and FLAG-PER purification, our ^15^N/^14^N quantitative MS did not yield satisfactory temporal resolution of PER PTM cycling. We therefore consolidated our MS data from multiple time-points with the goal of identifying PER O-GlcNAcylation sites qualitatively (Fig 1 and Table 1). We observed that multiple residues located in the CBD and within the CLK:CYC inhibition domain (CCID) [31, 32] are O-GlcNAcylated. These include PER(S942) as well as a potential sites at S951, T952, or T954. Our MS analysis yielded O-GlcNAc-modified peptides in which only PER(S942) is modified, providing unambiguous identification of PER(S942) as an O-GlcNAcylated residue (Fig 1 and Table 1). In addition, O-GlcNAc-modified peptides spanning S942 to T954 were also identified, but we were not able to narrow down the single modified residue within these peptides. It is possible that only S942 is O-GlcNAcylated within this region. Alternatively, either S951, T952, or T954 may represent a second O-GlcNAcylated residue within this region. Taken together, we hypothesize that O-GlcNAcylation in the CBD may be important in modulating PER-CLK interaction and PER repressor activity. In addition to the O-GlcNAcylated residues in the CBD, a number of other potential sites were identified in other parts of the PER protein, but most of them are not located in characterized functional domains.

**Table 1:** Identification of PER O-GlcNAcylation and phosphorylation sites in fly head tissues.

Peptide sequence with modified residue (underlined)	Mascot score	Amino acid position (only 1 residue is modified within aa in square brackets)	PTM
VSDSAYSNSCSNSQSQR	112	S-25	P
VSDSAYSNSCSNSQSQR	82	S-27	P
LSGSHSSGSSGYGGKPSTQASSSDMIIK	[98; 91; 89]	[S-47; S-48; S-45]	P
STSLEGENLYFQGGRDEEKPRPS	109	S-97	P
EQLQQEEEEDQSGSESEADRV	85	S-149	P
EQLQQEEEEDQSGSESEADRV	67	S-149 & S-151	2P
AKSEAAQSFPIPSPL	[83; 83; 83]	[S-164; S-169; S-174]	GlcNAc
AKSEAAQSFPIPSPL	58	S-164	P
AKSEAAQSFPIPSPL	56	S-174	P
ADLKLELPHENELTVSER	111	T-583	P
LELPHENELTVSER	49	S-591	P
DSVMLGEISPHHDYYDSK	96	S-596	P
SSTETPPSYNQLNYNENLLR	161	T-610	P
SGPMSPVHEGSGGSGSSGNFTTA	128	S-661	P
SNIHMSSVTNTSIA	65	[S-680; S-685; S-686; T-688; T-690; S-691]	GlcNAc
RGGSHSWEGEANKPK	89	S-826	P
RGGSHSWEGEANKPK	107	S-828	P
GGSHSWEGEANKPK	56	S-826 & S-828	2P
GAAGSAGGAVGTGGVGSGGAGVAGGGGSGTGVAGTPEGR	191	S-853	P
GAAGSAGGAVGTGGVGSGGAGVAGGGGSGTGVAGTPEGR	121	S-876	P
GAAGSAGGAVGTGGVGSGGAGVAGGGGSGTGVAGTPEGR	91	T-883	P
GGAVGTGGVGSGGAGVAGGGGSGTGVAGTPEGRAT	[43; 43; 42; 42; 42; 42]	[T-883; T-889; T-860; S-865; S-876; T-878]	GlcNAc
NLWPPFSVGITPPVHSTHTA	[119; 119; 119; 119; 199]	[S-942; S-951; T-952; T-954]	GlcNAc
NLWPPFSVGITPPVHST	51	S-942	GlcNAc
SLTPTSPTRSPR	[48; 43; 38]	[T-980; S-981; T-983]	P
SVYTTTPASMTK	[65; 65; 65; 65; 65; 65]	[S-1072; T-1075; S-1076; T-1077; S-1080; T-1082]	GlcNAc
KVPGAFHSVTTPAQVQRPSSQSASVK	103	S-1103	P
TEPGSSAAVSDPCKK	97	S-1119	P
VSDPCKKEVPDSSPIPS	[64; 62]	[S-1130; S-1129]	P
EVPDSSPIPSVMGDYNSDPPCSSSNPANNKK	[24; 24; 24; 24; 24; 24; 24]	[S-1129; S-1130; S-1134; S-1141; S-1146; S-1147; S-1148]	GlcNAc
TTDGSESPPDTEKDPK	101	S-1187	P
TTDGSESPPDTEKDPK	50	S-1185 & S-1187	2P

In addition to identifying PER O-GlcNAcylation sites, we took this opportunity to identify PER phosphorylation sites in fly tissues for the first time and confirm phosphorylation sites that have previously been identified in *Drosophila* S2 cells [17–19, 21]. Our analysis provides evidence that the majority of phosphorylation sites identified in *Drosophila* S2 cells are *bona fide* phosphorylation sites in fly tissues (Table 1). During the process of optimizing PER affinity purification and MS analysis using fly tissues as starting materials, we generated additional qualitative label-free MS datasets by analyzing PER phosphorylation sites at multiple time-points over the circadian cycle (ZT3, 16, 20, 24). S1 Table summarizes the label-free data and compares it to the phosphorylation sites identified in the ^15^N/^14^N-labeled MS analysis. PER O-GlcNAcylation site identification was not performed as part of the label-free MS analysis. We observed a high level of congruence between the PER phosphorylation sites identified in our two fly head tissue data sets, as well as those generated using *Drosophila* S2 cells as starting materials [17–19, 21]. In summary, our MS analysis sets the stage for future studies to understand the functional interplay between PER O-GlcNAcylation and phosphorylation.

### O-GlcNAcylation of PER regulates its repressor activity

**Fig 2:**
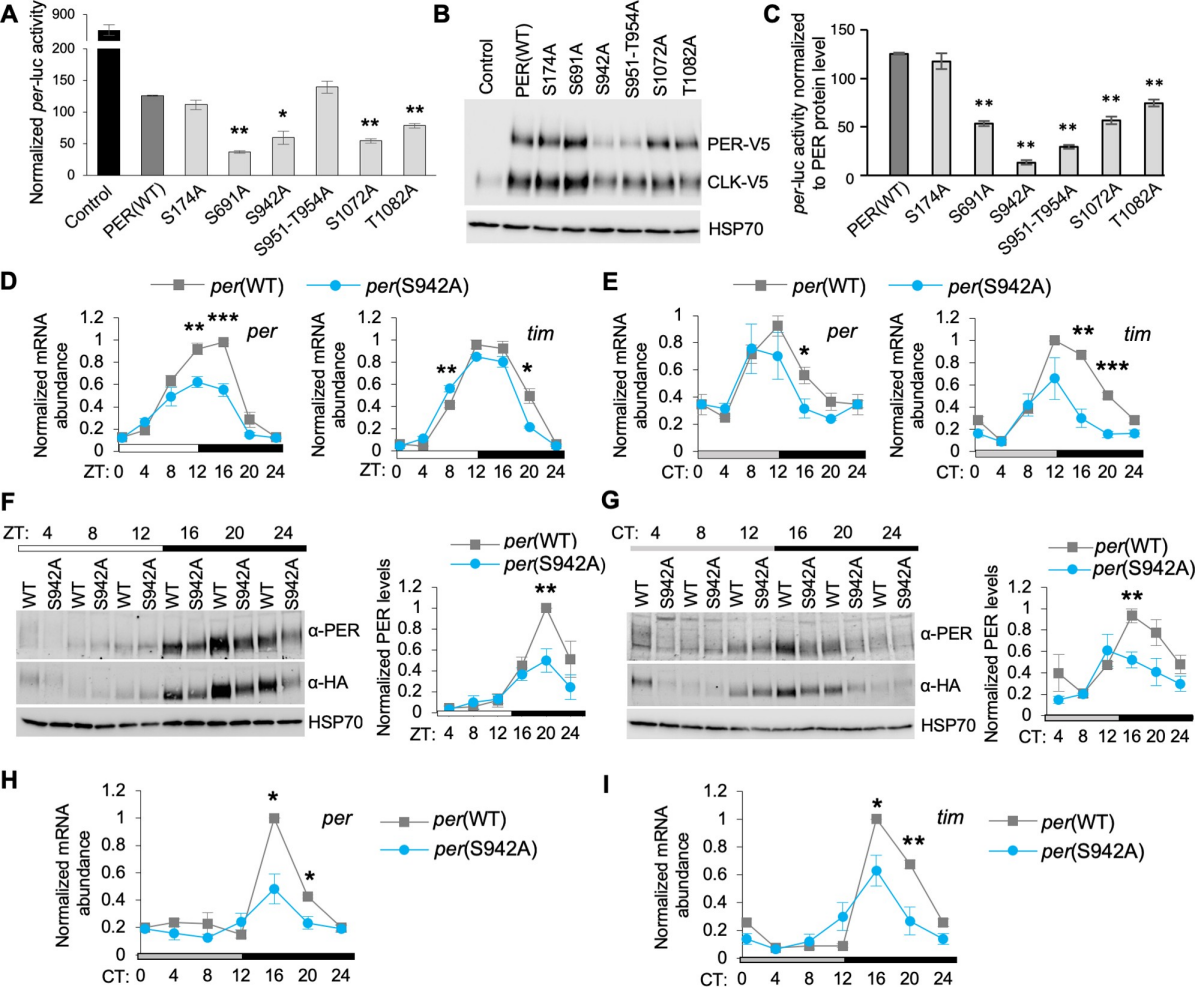
Eliminating O-GlcNAcylation at S942 elevates PER-dependent repression of CLK activity in *Drosophila* S2 cells and in flies. *(A)* Repressor activity of PER(WT) and PER O-GlcNAc site mutant variants as measured by *per-luc* reporter assay in S2 cells (n = 2). The control sample contains CLK in the absence of the repressor PER. Error bars indicate ± SEM. Asterisks denote statistical significance between WT PER and O-GlcNAc site mutants *P < 0.05, **P < 0.01; two-tailed Student’s t test. *(B)* Expression of WT and mutant variants of PER-V5 (Top) and CLK-V5 (Middle) in S2 cells for *per-luc* reporter assay. HSP70 was detected as loading control (Bottom). *(C)* Repressor activity of PER(WT) and PER O-GlcNAc site mutant variants as measured by *per-luc* reporter assay in S2 cells normalized to protein level of PER variants (n = 2). The control sample is not included as PER is not expressed. Error bars indicate ± SEM. Asterisks denote statistical significance between PER(WT) and O-GlcNAc site mutants **P < 0.01; two-tailed Student’s t test. *(D*,*E)* Steady state mRNA expression of *per* and *tim* in heads of *wper*^0^; *p*{*per*(WT)-HA10HIS} and *wper*^0^;*p*{*per*(S942A)-HA10HIS} flies, entrained in 12h:12h LD condition and assayed on LD3 (*D*) or DD1 (*E*) (n = 3 biological replicates). Error bars indicate ± SEM (*P-value < 0.05, **P-value < 0.01). *(F*,*G)* Western blots and corresponding quantifications comparing PER levels between head extracts of *wper*^0^; *p*{*per*(WT)-HA10HIS} and *wper*^0^; *p*{*per*(S942A)*-*HA10HIS} flies on LD3 (*F*) and DD1 (*G*). PER-HA levels were detected using both α-PER (GP5620) (Top) and α-HA (Middle). α -HSP70 was used to indicate equal loading and for normalization (Bottom). Three biological replicates were quantified and depicted in graphical format. Error bars indicate ± SEM (*P-value < 0.05, **P-value < 0.01). *(H*,*I)* Steady state mRNA expression of *per* (*H*) and *tim* (*I*) in fat body of *wper*^0^; *p*{*per*(WT)*-*HA10HIS} and *wper*^0^; *p*{*per*(S942A)-HA10HIS} flies, entrained in 12h:12h LD condition and assayed on DD1 (n = 3 biological replicates). Error bars indicate ± SEM (*P-value < 0.05, **P-value < 0.01).

Subsequent to the identification of PER O-GlcNAcylation sites, we proceeded to analyze the function of site-specific PER O-GlcNAcylation by mutating one or a cluster of S/T residues to non-O-GlcNAcylatable alanine on the *per* gene and evaluated PER repressor function on CLK activity using the *per-luciferase* (*per-luc*) reporter assay in *Drosophila* S2 cells [3, 37]. To prioritize, we analyzed PER O-GlcNAcylation residues with a Mascot score >50 (Table 1). We compared *per*-*luc* activity in S2 cells expressing *per* wild type or mutant variants (Fig 2A). In order to take into account the varying expression level of the PER variants (Fig 2B top panel, S3A and S3B Fig), we normalized CLK-activated *per-luc* activity observed for PER(WT) and PER mutant variants to their respective protein expression levels to more accurately assess the impact of blocking PER O-GlcNAcylation at specific residues on repressor activity (Fig 2C). We observed that all but one PER O-GlcNAcylation site mutant (PER(S174A)) exhibited significant increase in repressor activity. Interestingly, PER(S942A) and PER(T951A-S954A) are the two O-GlcNAc site mutants that exhibited the strongest repressor activity, further strengthening the rationale for testing the hypothesis that O-GlcNAcylation in the CBD may be important in modulating PER-CLK interaction and PER repressor activity. For this reason, this current study will focus on investigating the function of PER O-GlcNAcylation in the CBD, while analysis of other PER O-GlcNAcylation residues will be pursued in the future.

To confirm this finding in whole animals, we generated transgenic flies expressing *p*{*per*(WT)-HA10HIS} (herein referred to as *per*(WT) and mutant variants) in the *per*^0^ genetic background [38] so that only transgenic *per* (WT or mutant) was expressed. First, we performed quantitative RT-PCR to measure clock gene expression (*per* and *tim*) in heads of *per*(S942A) flies to examine the function of PER(S942) O-GlcNAcylation in central clock oscillators. All flies used for molecular analysis are homozygous for the *per* transgene. As predicted from the elevated PER repressor activity observed in S2 cells, we found that cycling of *per* and *tim* mRNAs was significantly dampened in *per*(S942A) flies as compared to *per*(WT) flies in both LD cycles and constant darkness (DD) (Fig 2D and 2E). Furthermore, *per* and *tim* mRNAs exhibited earlier initiation of repression phase in DD (Fig 2E). Since the *per*(S942A) mutant exhibited lowered levels of clock gene mRNAs, PER protein level is expected to decrease. As expected, peak PER abundance was significantly reduced in the heads of *per*(S942A) flies than in *per*(WT) flies in both LD and DD conditions (Fig 2F and 2G). To ensure that lower level of PER protein is a result of increased PER(S942A) repression on CLK-dependent transcription of *per* rather than reduced PER stability, we monitored the rate of PER degradation by cycloheximide (CHX) chase assay in S2 cells. The PER(S942A) mutant degraded at a similar rate as PER(WT) in the presence of OGT, demonstrating that the S942A mutation has no significant effect on PER stability (S3C and S3D Fig).

Since peripheral tissues are known to be more sensitive to metabolic fluxes [39, 40] and O-GlcNAcylation is a nutrient-sensitive PTM, we also analyzed the effect of blocking PER(S942) O-GlcNAcylation in oscillators of peripheral tissues, specifically the fat body. The fat body, which is analogous to mammalian liver and adipose tissue, plays an essential role in regulating energy metabolism in insects [39, 40]. We assayed *per* and *tim* mRNA levels in the fat body of *per*(WT) and *per*(S942A) flies on DD1 after LD entrainment to evaluate PER-dependent repression of CLK activity. Consistent with what we observed in fly heads, *per*(S942A) flies displayed significantly dampening of *per* and *tim* mRNA cycling as compared to *per*(WT) flies in the fat body (Fig 2H and 2I). This suggests that PER(S942) O-GlcNAcylation normally weakens the activity of PER to repress CLK-dependent transcription in both head and fat body oscillators.

As in PER(S942A) mutant, PER(S951A/T952A/T954A) mutant also exhibited a significant difference in repressor activity as compared to PER(WT) in *per-luc* reporter assay (Fig 2C). To evaluate the effects of these residues, which are also located in the CBD, we generated *wper*^*0*^; *per*(S951A/T942A/T954A) transgenic flies to confirm the effects of the PER(S951A/T952A/T954A) mutations in whole animals. We measured temporal cycling of *per* and *tim* mRNAs in heads of *per*(S951A/T952A/T954A) flies and observed dampened expression of *per* and *tim* mRNA as compared to *per*(WT) flies in both LD and DD conditions (S4A and S4B Fig), although not to the extent observed in *per*(S942A) flies. However, this did not translate into significant differences in PER protein abundance and cycling (S4C and S4D Fig). The differential effects observed when blocking O-GlcNAcylation at PER(S942) and PER(S951/T942/T954) suggest that PER(S942) O-GlcNAcylation is a key event in regulating PER repression within the CBD. It is important to stress that PER(S942) has never been identified as a phosphorylation site in all previous comprehensive mapping studies [17–19, 21], so it is highly unlikely that the effects for S942A mutation is due to disruption of PER(S942) phosphorylation.

### Site-specific inhibition of PER O-GlcNAcylation modulates locomotor activity and feeding rhythms in flies

**Fig 3:**
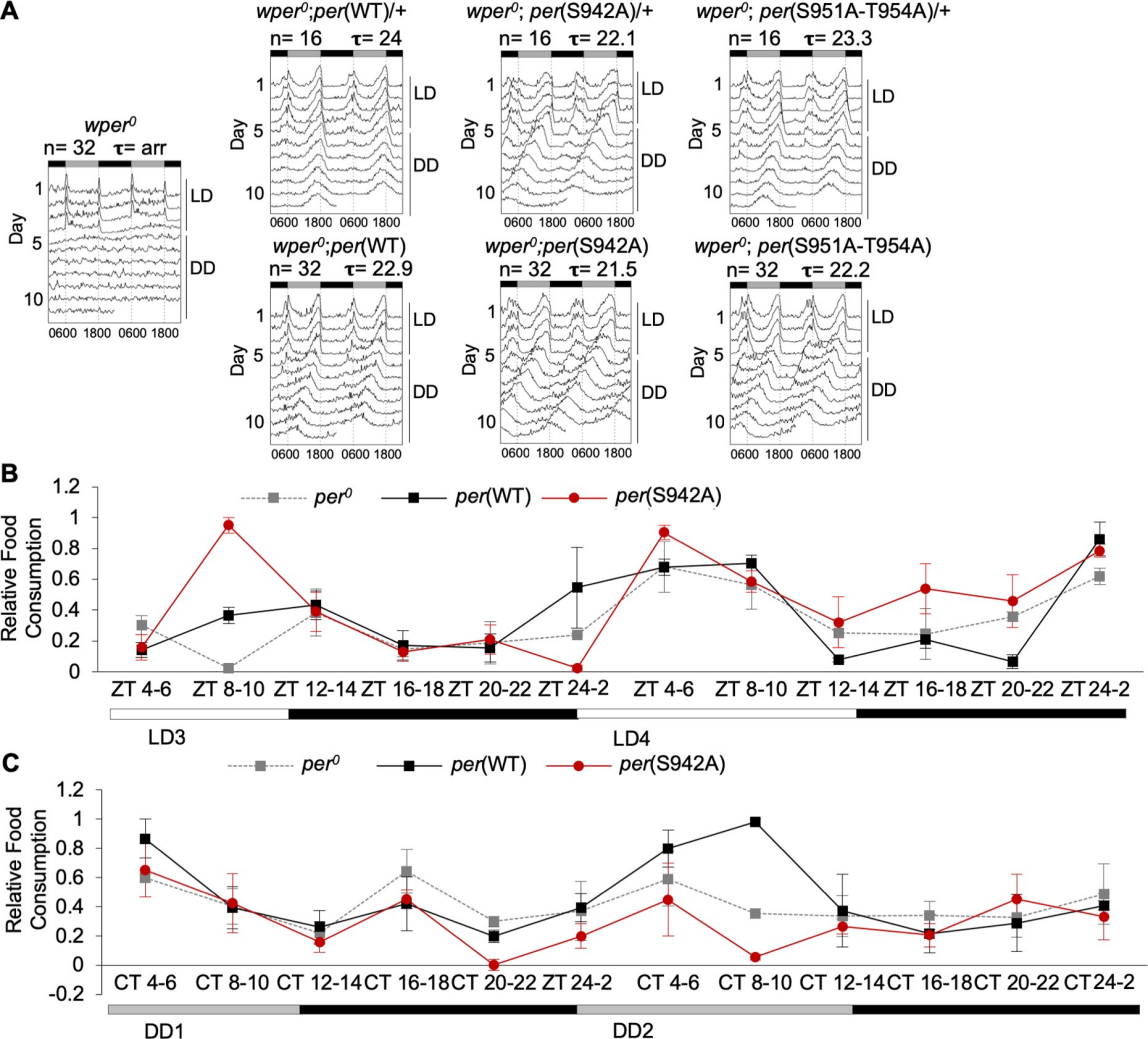
Locomotor activity and feeding rhythms of site-specific PER O-GlcNAcylation mutants are impaired. *(A)* Double-plotted actograms of *wper*^*0*^ flies carrying one copy or two copies of the *per* transgene (wild-type or O-GlcNAc *per* mutant). n represents sample size for locomotor activity assays. Tau (τ) represents average period length. *(B*,*C) wper*^0^; *p*{*per(WT)-*HA10HIS} and *wper*^0^; *p*{*per(S942A)-*HA10HIS} flies were entrained in 12h:12h LD condition and their feeding rhythms were assayed for two consecutive days of LD *(B)* or DD *(C)* by CAFE assay (n = 3). Error bars indicate ± SEM. Significant rhythms of feeding activity were observed in *wper*^0^; *p*{*per(WT)-*HA10HIS} and *wper*^0^; *p*{*per(S942A)-*HA10HIS} flies in LD (*P* < 0.01) but not in *per*^*0*^ flies as determined by JTK-cycle.

Given that several of the non-O-GlcNAcylatable *per* mutants exhibited elevation in PER repressor activity in S2 cells and in flies, we proceeded to investigate if this molecular phenotype can translate to alterations in output behavioral rhythms. We first evaluated daily locomotor activity rhythms of *per*(WT) and mutant flies. Activity rhythm is a robust readout that reflects the function and speed of the central oscillators located in fly heads [30]. Flies were entrained for 3 days in LD cycles followed by 7 days in DD. As expected, heterozygous *per*(WT) flies manifested robust rhythms with ~24-hr periods, indicating full rescue of arrhythmic *per*^*0*^ mutation (Fig 3A). Homozygous *per*(WT) flies displayed somewhat shorter behavioral rhythms due to the extra copy of *per*. Interestingly, as compared to *per*(WT), *per*(S942A) and the triple *per*(S951A/T952A/T954A) mutants homozygous for their respective transgenes exhibited shorter periods by 1.4 and 0.7 hrs. The earlier initiation of repression previously observed in *per*(S942A) mutant likely contributes to its short-period phenotype. In comparison, *per*(S951A/T952A/T954A) mutants displayed a smaller though significant change in repression activity, which likely accounts for the smaller change in period length.

In addition to monitoring locomotor activity rhythms of *per*(S942A) flies, we also examined the effects of blocking PER(S942) O-GlcNAcylation in peripheral oscillators by measuring feeding rhythms. Feeding assays were only performed to compare *per*(WT) and *per*(S942A) flies as *per*(S951A/T952A/T954A) mutants displayed minor changes in activity rhythms and clock gene repression. Feeding activity rhythms are governed by oscillators of metabolic tissues (i.e. in the fat body) [40], which are more sensitive to nutrient flux than central oscillators in the brain. Since we demonstrated earlier that clock gene cycling was dampened in the fat body of *per*(S942A) flies as a result of PER being a stronger repressor, we expected that feeding rhythms will also be disrupted. We entrained *per*(WT) and *per*(S942A) flies in 12h:12h LD cycles and assayed their feeding activity rhythms for two consecutive days either in LD or DD conditions using the CAFE assay [41]. The amount of food consumed over a 2-hr period was determined at 4-hr intervals over the circadian cycle. As control, *per*^*0*^ flies were measured in parallel, and as expected they exhibited arrhythmic feeding activity in LD and DD (Fig 3B and 3C) as determined by JTK-cycle (*P* = 1) [42].

*per*(WT) flies displayed robust rhythms of feeding behavior (JTK-cycle (*P <* 0.01)); they feed during daytime and fast at night in both LD and DD conditions (Fig 3B and 3C). Robust feeding rhythms were also observed in *per*(S942A) flies in LD (JTK-cycle (*P <* 0.01)), but the peak was phase advanced. This is consistent with the shorter period length in locomotor activity observed in the *per*(S942A) mutant. The consequence of blocking O-GlcNAcylation at PER(S942) on feeding behavior is even more severe in DD in the absence of light cues as rhythmic feeding was abolished in DD (JTK-cycle (*P* = 1)). Together, our data demonstrates that O-GlcNAcylation at PER(S942) modulates the function of central and peripheral oscillators by regulating PER activity.

### Timing of PER nuclear entry is not regulated by PER(S942) O-GlcNAcylation

Premature PER nuclear entry during the night could explain why *per*(S942A) mutant exhibits stronger and/or premature initiation of repression on CLK activity. A previous study showed that PER O-GlcNAcylation is involved in regulating nuclear entry [23]. To examine this possibility, we monitored timing of PER nuclear entry in adult clock neurons in LD cycles. In the fly brain, rhythmic expression and nuclear localization of PER in lateral clock neurons (LN_v_s) are necessary for proper clock function and maintenance of rhythmic locomotor activity (reviewed in [2]). The lateral neurons (small and large; l-LN_v_s and s-LN_v_s) express pigment dispersing factor (PDF), which has been commonly used to label the cytoplasm of the LN_v_s [43]. Despite having shorter behavioral rhythms, we found that *per*(S942A) mutants did not exhibit a significant difference in accumulation of nuclear PER as compared to *per*(WT) flies at time-points when nuclear entry is most prominent, ZT18 to ZT22 [4] (S5A and S5B Fig). Although a previous report demonstrates that global knockdown or overexpression of OGT in clock neurons affects the timing of PER nuclear entry [23], it is not surprising that site-specific non-O-GlcNAcylatable *per* mutants, in this case *per*(S942A), may not show the same phenotype. As such, our results suggest that the stronger and premature initiation of CLK repression observed in heads and fat bodies of short-period *per*(S942A) flies cannot be explained by altered timing of PER nuclear localization in circadian oscillators.

### PER(S942) O-GlcNAcylation is necessary for proper timing and level of repression by mediating PER-CLK interaction

**Fig 4:**
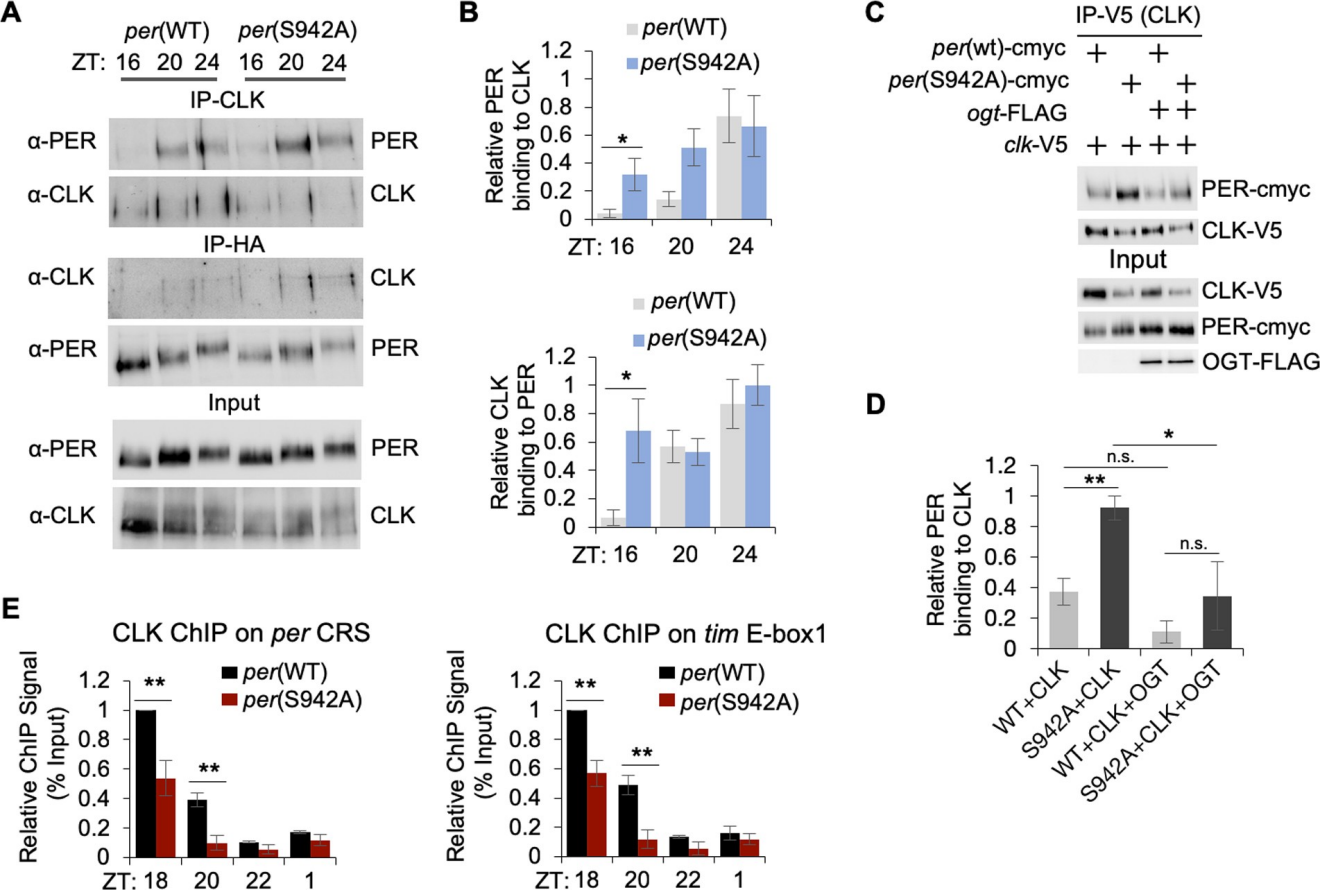
Non-O-GlcNAcylatable mutation at PER(S942) elevates PER repressor activity by increasing PER-CLK interaction and promoting CLK dissociation at circadian promoters. *(A)* Western blots showing reciprocal coimmunoprecipitations (coIPs) to examine PER-CLK binding in *wper*^0^; *p*{*per(WT)-*HA10HIS} and *wper*^0^; *p*{*per(S942A)-*HA10HIS} flies at the indicated time-points. Flies were entrained for 2 days in 12h:12h LD cycles and collected on LD3. Protein extracts from fly heads were directly analyzed (input) or immunoprecipitated with α-HA to detect PER-HA or α-CLK. Immune complexes were then subjected to immunoblotting to detect bait or interacting proteins. *(B)* Bar graphs showing quantification of reciprocal coIPs to examine PER-CLK binding from three biological replicates. Values for binding of interacting proteins are normalized to amount of bait detected in the IPs. Error bars indicate ± SEM. *P < 0.05. *(C)* Western blots showing results of coIP to evaluate PER-CLK interaction in S2 cells expressing pAc*-per*(WT)-6Xcmyc or pAc*-per*(S942A)-6Xcmyc with pAc*-clk*-V5 in the presence or absence of pMT*-ogt*-3XFLAG. Protein extracts from S2 cells were either directly analyzed by immunoblotting (input) or subjected to immunoprecipitation with α-V5 or resin conjugated with a non-specific antibody (α-HA) as negative control (no signal detected). Immune complexes were analyzed by α-V5 to detect CLK-V5 protein (bait) or by α-cmyc to assess interaction with PER-cmyc. *(D)* Bar graph shows quantification of PER normalized to IP-V5 (bait protein, CLK) from four independent experiments. Error bars indicate ± SEM. *P-value = 0.05, **P-value < 0.01. *(E)* ChIP assays using fly head extracts showing reduced CLK occupancy at *per* and *tim* promoters in *wper*^0^; *p*{*per(S942A*)-HA10HIS} as compared to *wper*^0^; *p*{*per(WT)-*HA10HIS} flies. Error bars indicate ± SEM (n = 3). *P-value < 0.05, **P-value < 0.01.

Given that PER does not enter the nucleus prematurely in the LN_v_s of *per*(S942A) mutant flies, we speculate that the PER(S942) may have a higher affinity to CLK that results in stronger and/or premature initiation of repression. We therefore examined PER-CLK interactions by performing co-immunoprecipitations (coIP) using head extracts from *per*(WT) and *per*(S942A) flies. These experiments were performed in head tissues as IP reactions of clock proteins are more efficient in head tissues and produce more robust results. Indeed, the *per*(S942A) flies showed significantly higher PER-CLK interaction at ZT16 than *per*(WT) flies (Figs 4A, 4B and S6A). To validate our results, we performed coIPs using S2 cells coexpressing *per*(WT)-cmyc or *per*(S942A)-cmyc with *clk-*V5, in the presence or absence of pMT-*ogt*-FLAG. Consistent with the fly data, we observed that PER(S942A) proteins exhibited significantly stronger binding to CLK as compared to PER(WT) (Fig 4C and 4D). Interestingly, when PER(S942A) mutant was coexpressed with CLK in the presence of OGT, the level of PER-CLK binding was lower than the levels when PER(S942A) mutant was coexpressed with CLK in the absence of OGT. This suggests that O-GlcNAcylation of CLK or perhaps at other PER residues may negatively impact PER-CLK interaction. Nevertheless, our results suggest that stronger and/or premature initiation of repression phase in *per*(S942A) flies is due to higher affinity between CLK and PER(S942A).

In comparison to *per*(S942A) mutant flies, *per*(S951A/T952A/T954A) flies only displayed minor changes in activity rhythms and clock gene expression in flies. Based on these observations, the PER(S951A/T952A/T954A) mutant protein is predicted to exhibit minor changes on PER-CLK interactions, despite that these three residues are also localized within the CBD. As expected, no significant changes in PER-CLK interactions were observed between *per*(WT) and *per*(S951A/T952A/T954A) flies at the indicated time-points (S7A and S7B Fig). Thus, our results suggest that O-GlcNAcylation events at PER(S951/T952/T954) only have minor modulatory effects on PER-CLK interactions. Alternatively, it is possible that only PER(S942) is O-GlcNAcylated within the CBD (Fig 1).

Since part of PER repressor function is to remove CLK from clock gene promoters, we would expect that CLK may be removed prematurely when PER(S942) O-GlcNAcylation is blocked. We therefore measured CLK occupancy at the E-box elements of *per* and *tim* promoters by performing chromatin immunoprecipitation (CLK-ChIP) using extracts from adult fly heads. Consistent with our hypothesis, we found that flies expressing *per*(S942A) showed significantly reduced CLK occupancy on *per* and *tim* promoters at ZT18 and ZT20 as compared to *per*(WT) flies (Fig 4E). To rule out the possibility that decreased CLK occupancy observed at ZT18 and ZT20 in *per*(S942A) flies was due to decreased CLK levels, we examined CLK levels after the ChIP reactions and found that CLK was not limiting in flies expressing *per*(WT) or *per*(S942A) (S6B Fig).

### OGT overexpression delays the timing of PER-CLK interaction

**Fig 5:**
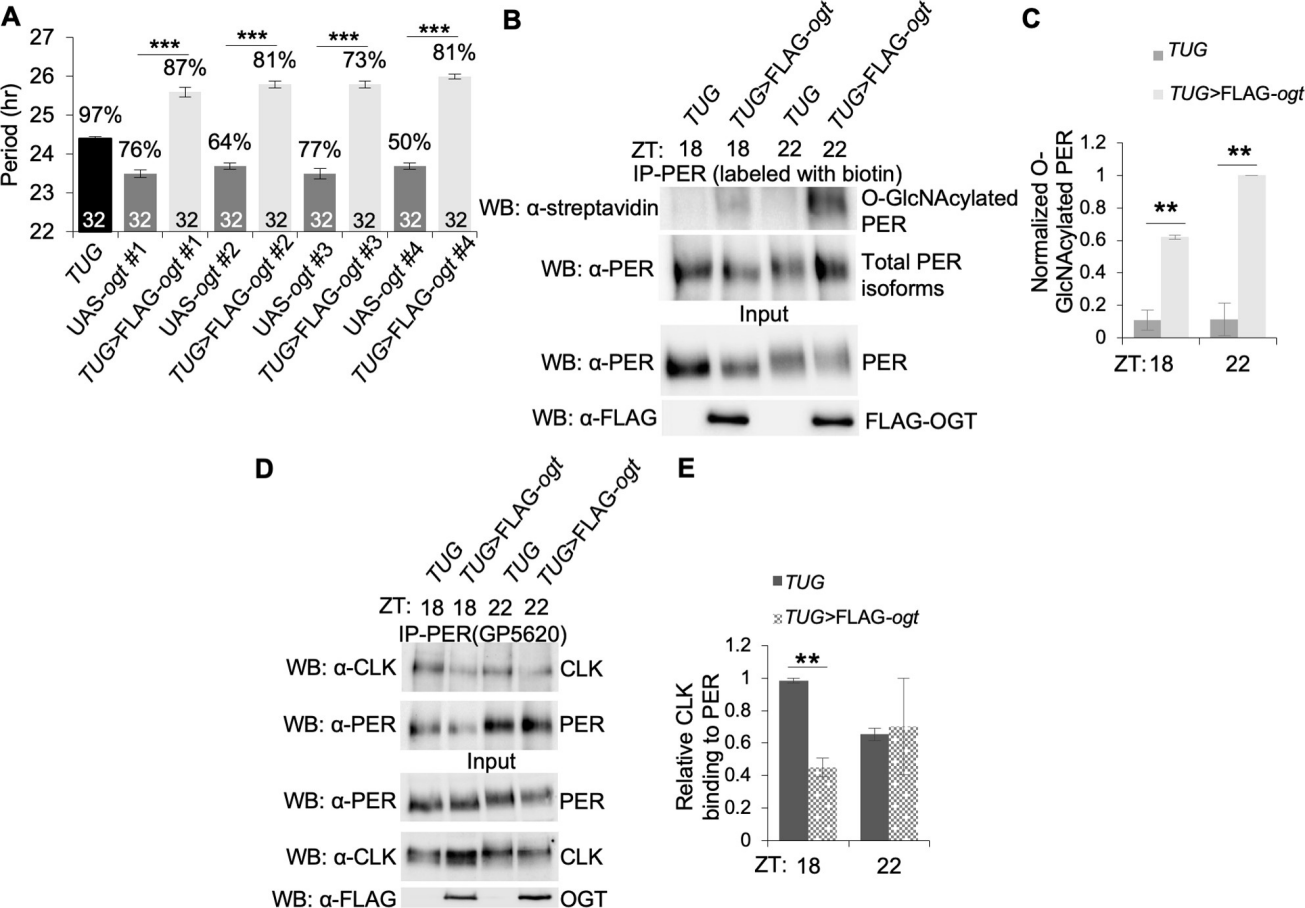
OGT overexpression in clock neurons lengthens behavioral rhythms and disrupts PER-CLK interaction. *(A)* Four independent transgenic fly lines expressing 3XFLAG-tagged *ogt* in *tim-*expressing neurons (*TUG>*FLAG*-ogt*) display lengthened periods in free-running locomotor activity rhythms as compared to their parental controls. Black and dark grey bars represent control parental lines. Light grey bars represent independent *TUG>*FLAG*-ogt* transgenic lines. Numbers above bars indicate percentage of flies displaying rhythmic activities. Asterisks above percentage denote statistical significance (***P-value < 0.001) in period lengths of transgenic flies expressing *TUG* >FLAG-*ogt* as compared to their parental controls. Numbers within bars indicate sample size (number of flies). Error bars indicate ± SEM. *(B)* PER O-GlcNAcylation is enhanced in *TUG>*FLAG*-ogt* (line 4 is used for all subsequent experiments). Protein extracts from fly heads at the indicated time-points were either directly analyzed by immunoblotting (input) (Bottom two panels) or immunoprecipitated with α-PER (GP5620), followed by chemoenzymatic labeling with biotin. Samples were resolved on SDS-PAGE and analyzed by western blotting using α-streptavidin or α-PER (Top two panels). *(C)* Bar graph showing normalized O-GlcNAcylated PER in *ogt* overexpressor and control flies (n = 2). Error bars represent SEM. **P = 0.01; two tailed Student’s t test. *(D)* Western blots showing reduced PER-CLK binding in *ogt* overexpressor flies at ZT18 but not at ZT22 as compared to control flies. *(E)* Bar graph showing quantification of CLK immunoprecipitated by α-PER (GP5620) (n = 2). Error bars = SEM. **P = 0.01; two tailed Student’s t test.

To further support our hypothesis that dynamic O-GlcNAcylation at PER(S942) is critical for regulating the strength and/or timing of PER-dependent clock gene repression, we overexpressed 3XFLAG-*ogt* in *tim-*expressing clock neurons using a *tim(UAS)*-*gal4* driver (referred to as *TUG*) [44] and examined PER-CLK interactions in head extracts of these flies. First, we verified that *ogt* overexpressors (*TUG*>FLAG-*ogt*) exhibited a significant ~2-hr period-lengthening of behavioral rhythms, as previously observed [23, 24] (Fig 5A). All 4 independent *ogt* overexpressor fly lines we generated showed similar period-lengthening phenotypes as compared to parental controls. To confirm that the overexpressed OGT enzyme is functional, we optimized O-GlcNAc chemoenzymatic labeling to measure levels of O-GlcNAcylated proteins in fly head extracts.

Currently, detecting protein O-GlcNAcylation remains to be a challenge when using traditional protein analytical techniques because the addition of a sugar group does not influence the migration of a polypeptide in gel electrophoresis (reviewed in [45]). Additionally, commercially available O-GlcNAc-specific antibodies or lectin yield non-specific signals in our hands. Thus, we chose to use a more sensitive and specific chemoenzymatic labeling method to examine *in vivo* protein O-GlcNAcylation status in this study [46–48].

We first tested the specificity of this approach by detecting O-GlcNAcylated PER and OGT in *Drosophila* S2 cell culture. S2 cells were transiently transfected with V5 tagged *per* with or without pMT-*ogt*-FLAG. Immunoprecipitated PER and OGT were labeled with resolvable mass tags prior to SDS-PAGE and western blotting. Results showed successful labeling of O-GlcNAcylated PER with 20 kD PEG mass tag in the presence of OGT, leading to clear mobility shift of O-GlcNAcylated PER isoforms (S8A Fig). As OGT is known to be auto-O-GlcNAcylated, addition of 10kD PEG mass tag in the reaction resulted in O-GlcNAcylated OGT isoforms that appeared as slower migrating bands as detected by Western blots (S8B Fig). The use of PEG mass tag labeling provides two advantages. First, both modified and non-modified isoforms can be detected, providing information on stoichiometry. Second, epitope-tagged antibodies or target-specific antibodies can be used for detection of all isoforms. Alternatively, O-GlcNAcylated proteins can also be labeled with biotin to facilitate detection with α-streptavidin antibodies [46–48]. Our success in detecting and visualizing *in vivo* O-GlcNAcylation status of proteins in S2 cells by chemoenzymatic labeling allowed us to proceed and confirmed that PER O-GlcNAcylation was elevated in head extracts of *ogt* overexpressor flies, an indication that overexpression of *ogt* led to increase in enzyme activity (Fig 5B and 5C).

Since blocking PER(S942) O-GlcNAcylation promotes PER-CLK interaction, we expect that PER-CLK interaction should be reduced in *ogt* overexpressors assuming PER(S942) is hyper-O-GlcNAcylated. As anticipated, we observed that control *TUG* flies exhibited significantly higher PER-CLK interaction at ZT18 as compared to *ogt* overexpressor flies in coIP assays using head extracts of flies (Fig 5D and 5E). For *ogt* overexpressor flies, the reduction in PER-CLK interaction observed in fly heads may partially account for the physiological effect of OGT overexpression, i.e. period-lengthening of clock-controlled locomotor activity rhythms (Fig 5A).

### Daily rhythms in PER O-GlcNAcylation correlate with feeding rhythms and suggest that PER(S942) O-GlcNAcylation prevents premature initiation of circadian repression

We speculate that PER(S942) O-GlcNAcylation occurs either during the daytime or early night to prevent *de novo* PER from prematurely binding to CLK or at late night to facilitate CLK dissociation from PER after PER-dependent repression. Both of these scenarios would result in stronger clock gene repression in *per*(S942A) flies. Since we observed that the differences in PER-CLK interactions between *per*(WT) and *per*(S942A) flies as well as between WT *TUG* control and *ogt* overexpressor flies occurred at around the start of the circadian repression phase (i.e. ZT16 and ZT18 respectively; Fig 4B and 5E), we postulated that the former scenario may be more likely. Temporal data on PER(S942) O-GlcNAcylation status would certainly help to rule out one of the two scenarios. It is therefore unfortunate that we are not able to determine the timing of PER(S942) O-GlcNAcylation by MS. Kim et al. [23] and Kaasik et al. [24] have previously examined global PER O-GlcNAcylation status over the circadian cycle using anti-O-GlcNAc antibodies in combination with Western blotting, but their results were incongruent. Whereas Kim et al. observed that PER O-GlcNAcylation peaks at ZT20 [23], Kaasik et al. observed a peak in PER O-GlcNAcylation at ZT10 [24]. We therefore opted to profile global PER O-GlcNAcylation status by chemoenzymatic labeling to gain insights into the timing and function of PER(S942) O-GlcNAcylation.

**Fig 6:**
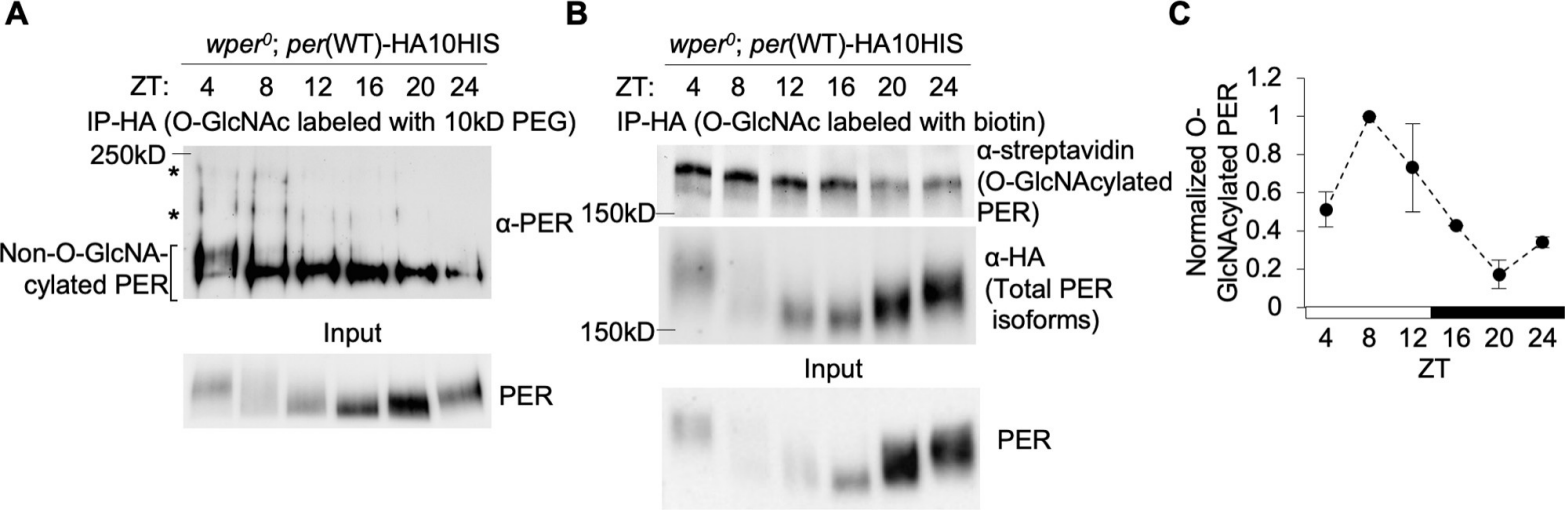
PER O-GlcNAcylation levels show correlation with feeding activity. *(A)* Western blot showing PER O-GlcNAcylation profile over a circadian cycle. PER proteins were immunoprecipitated by α-HA from head extracts of *wper*^0^; *p*{*per(WT)-*HA10HIS} flies entrained in 12h:12h LD cycle. O-GlcNAcylated PER was chemoenzymatically labeled with 20-kDa PEG mass tag, resolved by SDS-PAGE, and immunoblotted using α-PER (GP5620) (Top). Slower migrating bands, denoted by asterisks, are O-GlcNAcylated PER while non-glycosylated PER migrates faster. Input for immunoprecipitation and labeling were also directly probed for PER (Bottom). *(B)* PER proteins were immunoprecipitated by α-HA from head extracts of *wper*^0^; *p*{*per(WT)-*HA10HIS} flies entrained in 12h:12h LD cycle. O-GlcNAcylated PER was chemoenzymatically labeled with biotin, resolved by SDS-PAGE, and immunoblotted using α-streptavidin (Top) and α-PER (GP5620) (Middle). Input for α-HA IP was immunoblotted with α-PER (Bottom). *(C)* Quantification of PER O-GlcNAcylation over a circadian cycle as shown in Fig 6B (n = 2). Normalization was performed using the signal for α-HA IP for PER-HA. Error bars = SEM. Rhythmicity of PER O-GlcNAcylation was confirmed by JTK-cycle (*P* < 0.0005).

We examined the temporal profile of PER O-GlcNAcylation in head extracts of *per*(WT) flies in LD condition by labeling PER with either 20kD PEG or a biotin tag [46–48]. In both cases, maximal PER O-GlcNAcylation occurred at around ZT4 to ZT8 with subsequent decline from ZT12 to ZT24 (Fig 6A and 6B). Our results were more in line with the temporal PER O-GlcNAcylation profile observed in Kaasik et al. [24]. Daily PER O-GlcNAcylation cycle was found to be significantly rhythmic (JTK cycle; *P* < 0.0005) (Fig 6C). Our results reveal that PER is more highly O-GlcNAcylated during the day. This suggests that PER(S942) is likely O-GlcNAcylated during the day and may persist into early night to prevent *de novo* PER from prematurely interacting with CLK to initiate the repression phase.

O-GlcNAcylation of cellular protein is sensitive to nutrient input [25, 49]. Since we observed that PER O-GlcNAcylation is higher during the day and gradually decreases over the circadian cycle, we expect that this temporal pattern may correlate to daily feeding activity. We measured feeding rhythms in *per*(WT) flies fed *ad libitum* using the CAFE assay [41]. The flies used for these assays were entrained simultaneously with flies used for PER O-GlcNAc labeling to better assess correlation between feeding activity and PER O-GlcNAcylation. We found that mixed sexes of *per*(WT) flies displayed rhythmic feeding that peaked during early day in LD condition (S9A Fig). Similar results were obtained when male and female *per*(WT) flies were housed and tested separately (S9B Fig). Furthermore, a separate experiment comparing feeding activity rhythms of *per*(WT) and *per*(S942A) flies also showed higher daytime feeding activity in *per*(WT) flies (Fig 3B). Taken together, our data suggest that nutrient flux via feeding activity provides time-of-day metabolic signals to the circadian oscillator via temporal O-GlcNAcylation of PER. Specifically, PER(S942) O-GlcNAcylation, which is expected to occur during the day and persist into early night, may prevent interaction of PER and CLK prematurely to regulate timing of circadian repression.

## Discussion

Recent studies reveal that O-GlcNAcylation of circadian transcription factors, PER and CLK in *Drosophila* and BMAL1 and CLOCK in mice, plays an important role in modulating their function in the circadian oscillator [23–25, 50]. However, to more fully understand the mechanisms by which site-specific O-GlcNAcylation events regulate circadian physiology and to set the stage for investigating the interplay between phosphorylation and O-GlcNAcylation, it is necessary to identify O-GlcNAcylated residues in core clock proteins and other cellular proteins and characterize their site-specific functions. Furthermore, the relationship between feeding-induced nutrient influx and the temporal regulation of clock protein O-GlcNAcylation warrants investigation in whole animals. Since feeding activity is controlled by the circadian clock, we hypothesize that food intake will increase HBP influx, leading to increase in clock protein O-GlcNAcylation during the feeding period or soon after. This could serve as a mechanism by which metabolic input reinforces circadian entrainment by other zeitgebers and regulates oscillator function in a time-of-day specific manner. We therefore set out to (i) identify PER O-GlcNAcylation sites and characterize their site-specific functions in regulating the circadian oscillator; and (ii) determine if there is a correlation between time of feeding and O-GlcNAcylation levels of PER. Although overexpression or knockdown of OGT and OGA has provided insights into the global effects of protein O-GlcNAcylation on circadian clock regulation, we expect that site-specific characterization of O-GlcNAcylation events will alleviate confounding effects resulting from having multiple O-GlcNAcylated residues within a single protein or the involvement of multiple O-GlcNAcylated proteins in clock regulation, leading to new mechanistic insights.

**Fig 7:**
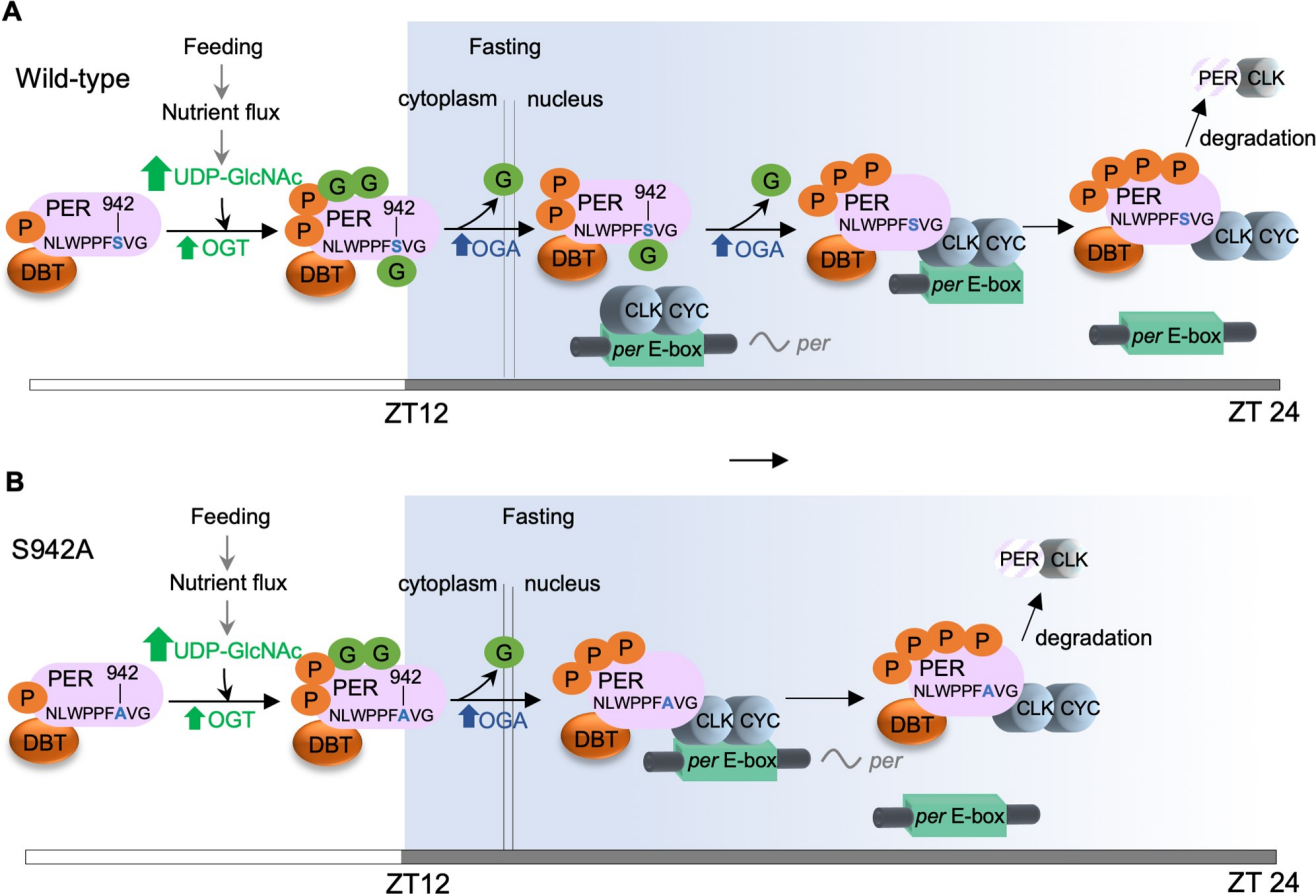
Model illustrating metabolic regulation of PER function in the *Drosophila* circadian clock through O-GlcNAcylation of Serine 942. *(A)* In wild-type flies, daily cycles of PER O-GlcNAcylation, including at S942, correlates with clock-controlled feeding rhythm. Nutrient intake during the day increases PER(S942) O-GlcNAcylation to prevent *de novo* synthesized PER from binding to CLK prematurely upon entry into the nucleus. O-GlcNAcylation of other PER residues may control other aspects of PER metabolism. e.g. nuclear translocation [23]. OGA-dependent removal of O-GlcNAc from unknown residue(s) allows PER to enter the nucleus in early night. Thereafter, removal of O-GlcNAc at S942 by OGA enables PER to interact with CLK to repress CLK-mediated clock gene transcription. *(B)* In flies expressing *per*(S942A), PER nuclear translocation is not affected. Upon nuclear entry, PER binds to CLK earlier and triggers a phase advance by repressing CLK activity and removing CLK from target promoters prematurely. The white/dark grey bar on the bottom of each panel represents the light/dark period of the circadian cycle. Nutrient influx and feeding/fasting period are indicated. G denotes O-GlcNAc; P denotes phosphorylation.

By utilizing MS-based proteomics, we observed that in addition to being heavily phosphorylated, PER is O-GlcNAcylated at at least 6 residues, some of them in the CBD. To understand the role of these O-GlcNAcylation events in regulating PER function and circadian physiology, we analyzed these O-GlcNAcylation sites by replacing serine or threonine to non-GlcNAcylatable alanine either singly or in clusters. Several of these non-O-GlcNAcylatable *per* mutants exhibit changes in PER repressor function, which consequently result in period-changing phenotypes in their corresponding transgenic fly lines. In particular, we observed that O-GlcNAcylation at PER(S942), which is located in the CBD, reduces PER-CLK interaction (Fig 7A and 7B). Using O-GlcNAc chemoenzymatic labeling, we show that PER O-GlcNAcylation primarily occurs during daytime and correlates with the time period when animals are feeding. We therefore postulate that PER(S942), as in the case for most PER O-GlcNAcylation sites, is O-GlcNAcylated during the day. This ensures that PER does not interfere with CLK activity in the circadian activation phase and its repression of CLK activity does not initiate prematurely when *de novo* PER starts to translocate into the nucleus. This suggests that OGA may need to actively remove O-GlcNAc from PER residues prior to circadian repression phase. Indeed, OGA level has been shown to oscillate over the circadian day, peaking prior to initiation of circadian repression phase [24]. Moreover, O-GlcNAcylation has been shown to regulate PER nuclear entry [23], suggesting that OGA-dependent removal of O-GlcNAc at unknown PER residue(s) is likely required to facilitate PER nuclear translocation independent of OGA activity on PER(S942). Finally in the evening, since flies are fasting, the level of O-GlcNAcylation at PER(S942) will remain low allowing PER to bind strongly to CLK to repress its activity.

Our findings that PER O-GlcNAcylation at S942 reduces PER repression of CLK activity is not congruent to the observation in [24], where they observed that the repressor activity of PER is enhanced when coexpressed with OGT in S2 cell *per-luc* reporter assay. However, this apparent incongruence could be explained by the combined activities of other O-GlcNAcylation events on PER, CLK, or other cellular proteins that impact *per-luc* reporter gene expression. Nevertheless, it is important to point out that none of our non-O-GlcNAcylatable *per* mutants showed a decrease in repressor activity (Fig 2C).

It is interesting to note that unlike some phosphorylation sites previously identified on PER proteins, residues we identified to be O-GlcNAcylated in *Drosophila* PER are not conserved in mouse PER proteins. This is perhaps not surprising since nutrient-dependent O-GlcNAcylation on cellular proteins likely depends on when organisms are actively feeding, i.e. whether they are diurnal or nocturnal. For instance, mice are nocturnal, which may restrict protein O-GlcNAcylation of cellular proteins to nighttime. This nighttime peak in O-GlcNAcylation levels corresponds to when mouse PER2 proteins are abundant and active as repressors, suggesting that O-GlcNAcylation may act to promote PER2-dependent repression on circadian transcription. On the other hand, given that flies are diurnal and feed during the day (Fig 3B), nutrient flux promoting O-GlcNAcylation of *Drosophila* PER inhibits PER function as a transcriptional repressor (Fig 7). Overall, the differences in timing of feeding activity and nutrient flux between diurnal and nocturnal animals will present interesting opportunities for comparative analysis with regard to site-specific and global effects of O-GlcNAcylation on cellular protein function.

In addition to regulating PER-CLK interaction in the *Drosophila* circadian clock, O-GlcNAcylation is expected to affect oscillator function via other mechanisms [23–25]. Besides regulating clock proteins directly, it is likely that O-GlcNAcylation can modify the activity of clock kinases, just as GSK3β can regulate the activity of OGT [24]. CK2 and GSK3β are two clock kinases that have been shown to be substrates of OGT [51–52], and currently it is unclear how OGT might modulate their activities in clock regulation. Furthermore, O-GlcNAcylation can also impact the activities of chromatin modifiers and transcription machineries, including RNAPII [53]. The interplay between O-GlcNAcylation and other PTMs to regulate circadian physiology is expected to be complex and extensive, and reach far beyond the molecular oscillator and circadian transcription.

In summary, our results support that clock-controlled feeding activity provides metabolic input to reinforce entrainment signals by light-dark cycles to regulate circadian physiology via clock protein O-GlcNAcylation. We expect that circadian rhythms in peripheral systems, where oscillators are more sensitive to metabolic input, to be particularly sensitive to modulation via O-GlcNAcylation of clock proteins. Finally, our results imply that disruptions in daily feeding rhythms, e.g. irregular meal times and late night eating common in modern societies, will likely affect rhythms in protein O-GlcNAcylation and interplay with other PTMs, thereby disrupting circadian rhythms in physiology. Future experiments to manipulate feeding schedules by time-restricted feeding (TRF) [54] can further solidify the causal relationship between feeding-induced nutrient influx and O-GlcNAcylation of cellular proteins. They can also provide mechanistic insights into the benefits of TRF.

## Materials and methods

### Transgenic *Drosophila* construct design and fly transformation

To profile PER PTMs, we generated transgenic flies that expressed a 13.2 kb genomic clone of *per* in *w*^*1118*^ *per0 (wper*^*0*^*)* background. A previously characterized vector that contains a 13.2kb *per* genomic fragment tagged with HA and 10X histidine at the carboxyl terminal (*p*CaSpeR-*per(13*.*2WT)-*HA-10HIS) [55] was used as the template for inserting 3XFLAG at the amino terminal before the starting Methionine to facilitate FLAG Affinity Purification. Transformants were generated by P-element transformation (BestGene Inc., Chino Hills, CA), and the 3XFLAG-*per(13*.*2WT*)-HA-10HIS transgene was tested for functionality by determining whether it can rescue *wper*^*0*^ flies in behavioral and molecular assays.

To generate transgenic flies carrying wild-type (WT) or O-GlcNAcylation site mutants of *per*, we opted to use PhiC31 site-directed recombination [56]. The genomic *per*(13.2WT)-HA-10HIS was excised from *p*CaSpeR-*per(13*.*2WT)-*HA-10HIS using the restriction sites XhoI and BamHI and subcloned into *pattB* vector (kind gift from Amita Seghal) to yield *pattB*-*per*(13.2WT)-HA-10HIS. The *pattB* vector was modified so that the sites KpnI and XbaI were removed, the BgIII site was replaced by a BamHI site, and the BamHI site was replaced by a BgIII site. For generating flies expressing non-O-GlcNAcylatable mutants on *per*, a 3.4 kb XbaI-BamHI or 1kb BamHI-KpnI genomic fragment was excised from the p*attB*-*per*(13.2WT)-HA-10HIS plasmid and subcloned into a pGem7 vector [20]. The resulting pGem7-*per*(XbaI-BamHI) or pGem7-*per*(BamHI-KpnI) plasmid served as a parent template for site-directed PCR mutagenesis (Agilent Technologies, Santa Clara, CA) depending on the location of the O-GlcNAc site (S2 Table for mutagenic primer sequences). After mutagenesis and confirmation by Sanger sequencing (UC Davis Sequencing), the mutant variants of 3.4 kb or 1 kb *per* subfragments were used to replace the WT fragments in *pattB*-*per*(13.2WT)-HA-10HIS. Plasmids were injected into *w*^*1118*^ fly embryos carrying *attP* sites on chromosome 3 (*attP2*) (BestGene, Inc.). Transformants were crossed with *wper*^*0*^ flies to remove endogenous copies of *per* prior to behavioral and molecular analyses.

To generate flies overexpressing *ogt*, *ogt* was amplified and subcloned into pUAST*-attB* vector [57]. 3XFLAG was added to the N-terminus of *ogt* during the cloning process. Plasmids were injected into *w*^*1118*^ fly embryos carrying *attP* sites on chromosome 3 (*attP2*) (BestGene, Inc.). To express 3XFLAG-*ogt* in clock neurons, transgenic flies carrying the *UAS-*FLAG-*ogt* transgene were crossed to *w; tim-(UAS)-GAL4* (referred to as *TUG*) driver line [44].

### CAFE assay

CAFE assay was performed as described [41] with modifications. Mixed-sex population of five male and five female *wper*^*0*^, *wper*^*0*^*; per*(13.2WT), and *wper*^*0*^*; per*(S942A) flies or separately housed male or female (10 per group) flies were fed Bloomington *Drosophila* Stock Center standard fly food during entrainment in 12 h light/12 h dark day-night cycles and food consumption were measured starting on the third day of LD or the first day of DD. Prior to the day of measurement, grouped flies were transferred to a vial containing 2% agar as the medium with 5% sucrose solution maintained in calibrated glass micropipettes (VWR). After 24 hours of training, old micropipettes were replaced by fresh experimental micropipettes filled with 5% sucrose solution approximately 2 hours before each of the indicated time-points. After 2 hours, the amount of liquid consumed from the experimental micropipette was recorded, and the evaporation effect was evaluated by measuring the change in liquid volume in a micropipette placed in a vial without flies. Food consumption for each group/vial was determined by subtracting the amount of liquid consumed from the experimental micropipette with the amount of evaporated liquid. These values were normalized to the amounts of flies in the vial that survived until the end of the experiment. These experiments were performed in biological triplicates (one group/vial represents one independent experiment). Error bars = SEM. Rhythmicity of feeding was determined by JTK Cycle [42].

### Chemoenzymatic labeling of O-GlcNAc moiety and detection of *in vivo* O-GlcNAcylation status of target proteins

Proteins from S2 cells and fly heads were extracted using modified RIPA buffer as previously described [58]. Extracts were quantified and either directly analyzed by immunoblotting or incubated with 15μ α-V5 resin (Sigma) or 20μl of α-HA resin (Sigma) at 4°C for 4 hours. Beads were washed once in M-RIPA and twice with reaction buffer (20mM HEPES pH 7.9, 50mM NaCl, 1μM PUGNAc, 25mM NaF, 0.5mM PMSF, and 5mM MnCl_2_) supplemented with 1x protease inhibitor (Sigma) [59]. Procedures for chemoenzymatic labeling with biotin or PEG (Polyethylene Glycol) 10kD or 20kD mass tag were performed as described [48] with modifications. Attachment of biotin or PEG mass tag to O-GlcNAc group requires a two-step derivatization process [59]: (1) a mutant galactosyltransferase, GalT1 (Y289L), utilizes UDP-azidogalactose (UDP-GalNAz) as substrate to add an azide onto the O-GlcNAc group; (2) Biotin alkyne or an alkyne-functionalized PEG mass tag indirectly attaches to the O-GlcNAc group via azide-alkyne cycloaddition chemistry. Briefly, after immunoprecipitation followed by washes, immune complexes were resuspended in 20 μl of reaction buffer containing 2 μl of Gal-T1 Y289L (Invitrogen) and 2μl of 0.5mM UDP-GalNAz (Invitrogen) [59] following overnight incubation at 4°C with gentle rotation. Azide-labeled beads were washed twice with reaction buffer and subsequently resuspended in 50μl of labeling buffer (1% SDS and 50mM Tris-HCl pH 8.0). The samples were reacted with biotin alkyne (Invitrogen) or an alkynyl-functionalized poly(ethylene glycol) (10-kDa or 20-kD) (Creative PEGWorks, Chapel Hill, NC) according to manufacturer’s protocol or a previously described protocol by [59] respectively. Samples were eluted in 50μl of 1XSDS sample buffer. For proteins labeled with a mass tag (PEG), PER or OGT was resolved using SDS-PAGE (5% or 8% minigel with a 40:1 acrylamide/Bis-acrylamide solution, Bio-rad, Hercules, CA). For proteins labeled with biotin, PER was resolved using SDS-PAGE (5% Criterion gels, Bio-rad). Antibody dilutions to detect O-GlcNAcylated PER or OGT proteins are as follows: α-V5 (1:5000), α-HA (1:1000), α-FLAG (1:7000), α-streptavidin (Cell Signaling Technologies, Danvers, MA) (1:5000), and α-PER (GP5620; RRID:AB_2747405) (1:2000).

### Stable isotope labeling of *Drosophila* proteins and affinity purification of PER for ^15^N/^14^N-labeled proteomics

*wper*^*0*^; p{3XFLAG-*per(13*.*2WT*)-HA10HIS} flies were fed with an ^15^N diet (0.2g ^15^N yeast, 1% Bacto agar, 15% unsulfured molasses, phosphoric and propionic acid mix, and tegosept). *Saccharomyces cerevisiae* were metabolically ^15^N-labelled as described [29, 60]. As control, flies were also fed with ^14^N diet. The adult progenies of ^15^N- or ^14^N-fed parental flies were reared in ^15^N- or ^14^N diet and entrained for 3 days in 12hr light:12hr dark at 25°C and collected every four hours over a period of 24 hours on the fourth day. Upon collection, flies were immediately frozen on dry ice until protein extraction. For each time-point, 4ml of fly heads were homogenized into fine powder by a chilled mortar and pestle and resuspended in Lysis Buffer (20mM HEPES pH 7.9, 5% glycerol, 350mM NaCl, 0.1% Triton X-100, 1mM DTT, 1mM MgCl_2_, 0.5mM EDTA, 25mM sodium fluoride, 1x protease inhibitor (Sigma, St. Louis, MO), 1x PhosSTOP (Roche, South San Francisco, CA). Homogenate was dounced for 15 strokes using a 50ml tissue grind tight pestle (Kimble-Chase, Vineland, NJ) and were filtered using a 70μm cell strainer. Samples were spun at 300 rcf for 1 minute and then incubated at 4°C on a nutator for 30 minutes. Additional Lysis Buffer was added to dilute the sample from 350mM to 150mM NaCl before centrifuging at 15,000 rpm for 15 minutes at 4°C. Supernatant was collected and incubated with 200μl α-FLAG resin (Sigma) overnight over a nutator at 4°C. Next day, beads were washed twice for 15 mins with Lysis Buffer without EDTA, DTT, or PhosSTOP. Samples were eluted in 300ul R+A buffer (30% glycerol, 3% SDS, 6mM EDTA, 0.15M Tris-HCl pH 6.8) at 95°C. Eluate was reduced with 15μl 1M DTT for 10 minutes at 65°C and then alkylated with 35μl 1M IAA in room temperature for 20 minutes in the dark. Eluates were then flash frozen using liquid nitrogen immediately. We used the pooled standard approach to enable more accurate comparisons between different time points. ^14^N eluate from six time-points were pooled together and split evenly to mix with each ^15^N eluate at a 1:1 ratio on ice. For each time-point, 600μl of cold acetone was added to the ^14^N/^15^N eluate mixture and placed in -20°C overnight. Precipitate were spun at 14,000 rpm for 10 minutes at 4°C and the resulting pellet were washed briefly with 1ml cold acetone. Precipitated eluate was resuspended in 80μl R+A sample buffer containing 3μl of 4X SDS sample buffer. The ^14^N/^15^N eluate was resolved in 12% SDS-PAGE and the excised PER band was used for protease digestion and analysis by mass spectrometry.

### Processing of stable isotope labeled proteins for LC-MS/MS and data analysis

For analysis of ^14^N/^15^N-labeled samples, proteins were digested in-gel with trypsin and elastase in separate reactions to result in overlapping peptides, such that individual modified sites can be determined. We have previously used this multi-protease approach [61] to maximize high sequence coverage when mapping PER phosphorylation sites [17]. For in-gel digestion the excised gel bands were destained with 30% ACN, shrunk with 100% ACN, and dried in a Vacuum Concentrator (Concentrator 5301, Eppendorf, Hamburg, Germany). Digests with trypsin and elastase were performed overnight at 37°C in 0.05 M NH_4_HCO_3_ (pH 8). About 0.1 μg of protease was used for one gel band. Peptides were extracted from the gel slices with 5% formic acid.

NanoLC-MS/MS analyses were performed on an LTQ-Orbitrap Velos Pro or an Orbitrap Fusion (Thermo Fisher Scientific, Waltham, MA) equipped with an EASY-Spray Ion Source and coupled to an EASY-nLC 1000 (Thermo Fisher Scientific). Peptides were loaded on a trapping column (2 cm x 75 μm ID. PepMap C18, 3 μm particles, 100 Å pore size) and separated on an EASY-Spray column (25 cm x 75 μm ID, PepMap C18, 2 μm particles, 100 Å pore size) with a 90-minute linear gradient from 3% to 30% acetonitrile and 0.1% formic acid. For the Oribtrap Velos MS scans were acquired in the Orbitrap analyzer with a resolution of 30,000 at m/z 400, MS/MS scans were acquired in the Orbitrap analyzer with a resolution of 7,500 at m/z 400 using HCD fragmentation with 30% normalized collision energy. A TOP5 data-dependent MS/MS method was used; dynamic exclusion was applied with a repeat count of 1 and an exclusion duration of 30 seconds; singly charged precursors were excluded from selection. Minimum signal threshold for precursor selection was set to 50,000. Predictive AGC was used with AGC target a value of 1e6 for MS scans and 5e4 for MS/MS scans. Lock mass option was applied for internal calibration in all runs using background ions from protonated decamethylcyclopentasiloxane (m/z 371.10124). For the Orbitrap Fusion, both MS and MS/MS scans were acquired in the Orbitrap analyzer with a resolution of 60,000 for MS scans and 15,000 for MS/MS scans. HCD fragmentation with 35% normalized collision energy was applied. A Top Speed data-dependent MS/MS method applying HCD and ETD fragmentation from the same precursor with a fixed cycle time of 3 seconds was used. Dynamic exclusion was applied with a repeat count of 1 and an exclusion duration of 120 seconds; singly charged precursors were excluded from selection. Minimum signal threshold for precursor selection was set to 50,000. Predictive AGC was used with AGC a target value of 5e5 for MS scans and 5e4 for MS/MS scans. EASY-IC was used for internal calibration.

Mascot Distiller 2.5 was used for raw data processing and for generating peak lists, essentially with standard settings for the Orbitrap (high/high settings). Mascot Server 2.5 was used for database searching with the following parameters: peptide mass tolerance: 7 ppm, MS/MS mass tolerance: 0.02 Da, enzyme: “semi-trypsin” for tryptic digests and “none” for elastase digests; fixed modifications: carbamidomethyl (C); variable modifications: Gln->pyroGlu (N-term Q), oxidation (M), acetyl (protein N-term), phosphorylation (STY), HexNAc (ST). Searches containing both HCD and ETD spectra (Fusion) were searched separately for either b and y ions (HCD) or c and z ions (ETD). For ETD-searches different modifications definitions (without neutral losses) for phosphorylation and HexNAc were applied. Separate Mascot searches were performed for light peptides (quantitation: “none”) and heavy peptides (quantitation: ^15^N-labeling). Database searching was performed against a small custom database containing 187 of the most abundant proteins identified in these samples before in a first round search (without PTMs) against UniProt *Drosophila* database. This was necessary to limit search space and processing times.

The results from the different Mascot searches (different time-points, proteases and fragmentation techniques) were merged (separately for light and heavy peptides) and filtered for phosphorylated and HexNAc-modified peptides using a custom software tool (A. Schlosser). A Mascot score cut-off of 15 and a delta score cut-off of 10 [62] were applied, and only “rank 1” peptides were accepted. For one modification site and one type of modification, only the peptide spectrum match (PSM) with the highest score was kept, all other PSMs were filtered out. All remaining spectra were verified manually, e.g. by checking the presence of modification specific marker ions. HexNAc-modified peptides were only accepted when at least one of the HexNAc-specific fragment ions (204, 186 and 168) [63] was present in the corresponding HCD spectra. After manually filtering, all remaining peptides were exported to generate a summary of the results (Table 1). The N^14^/N^15^ MS data have been submitted to the Chorus repository (project ID 1424): (https://chorusproject.org/anonymous/download/experiment/e47a30f7f2c749aba438652d7d88ef04) and (https://chorusproject.org/anonymous/download/experiment/e6d6163b31bf40288606f827c6f18371).

### Affinity purification, sample and data processing for label-free mass spectrometry analysis

All flies were reared on standard *Drosophila* medium (Bloomington *Drosophila* Stock Center standard recipe). Entrainment and collection of flies at the appropriate time-points were described as above. Roughly 4ml of fly heads were grinded into fine powder using chilled ceramic mortar and pestle and mixed in 30ml of lysis buffer (20mM HEPES pH 7.5, 1mM DTT, 1x protease inhibitor). Homogenate was dounced and poured over a cell strainer as described above prior to centrifugation at 7000xg for 45 minutes at 4°C to separate nuclear and cytoplasmic lysates, repeated once. Lower layer (pellet) as the nuclear fraction from both spins was resuspended in 10ml Nuclear Extraction buffer (20mM HEPES pH 7.5, 10% Glycerol, 350mM NaCl, 0.1% Triton X-100, 1mM DTT, 1mM MgCl_2_, 0.5mM EDTA, 1x protease inhibitor, 10mM NaF) with the addition of MG132 (Sigma) and DNAse (Promega). Upper layer (supernatant) as the cytoplasmic fraction was supplemented with Lysis buffer with the addition of MG132 (Sigma) and DNAse (Promega). Nuclear and cytoplasmic fractions were incubated at 4°C for 30 minutes over a nutator. After incubation, nuclear fraction was diluted to 150mM NaCl with Lysis buffer. Nuclear and cytoplasmic fractions were centrifuged at 27,000rpm for 15 minutes at 4°C. Supernatant of nuclear and cytoplasmic samples was recovered before incubation with 200μl α-FLAG M2 resin (Sigma) at 4°C overnight. Beads were washed three times with Lysis buffer for 15 minutes each and subsequently eluted in 200μl of 3XFLAG peptide (Sigma) at a dilution of 250μg/ml at room temperature for 15 minutes. Eluates were resolved on a Tris-Tricine gel and PER bands were excised for protease digestion and mass spectrometry as described in [64]. The label-free MS proteomics data for PER phosphorylation site mapping have been deposited into ProteomeXchange (PXD008281) (ProteomeXchange: http://proteomecentral.proteomexchange.org/cgi/GetDataset?ID=PXD008281), MassIVE repository (MSV000081736) (MassIVE: https://massive.ucsd.edu/ProteoSAFe/dataset.jsp?task=384c7750b3004b7eac91054935a4e038), and Chorus repository (Project ID 1424) (https://chorusproject.org/anonymous/download/experiment/1e0023a15da84e51bb18c55146104b32).

### Measuring daily locomotor activity rhythms in flies

Locomotor activity rhythms were measured as previously described [30]. 3-4-day old male flies were collected and subjected to 12hr light:12hr dark (LD) cycles at 25°C for four days followed by seven days of constant darkness (DD) to measure free-running period using the *Drosophila* Activity Monitor System (DAMS) (TriKinetics, Waltham, MA). Data analysis was performed using FaasX as described in [30].

### Plasmids for S2 cell culture

pAc-*per*-V5, pAc-3XFLAG-*per*-6Xcmyc, and pAc-*clk*-V5 were previously described [20, 58]. For generating Serine/Threonine (S/T) to Alanine (A) O-GlcNAc site mutants (S2 Table for primer sequences), pAc-*per*-V5 or pAc-3XFLAG-*per*-6Xcmyc served as the template for site-directed PCR mutagenesis using QuikChange site-directed PCR mutagenesis (Agilent Technologies). All O-GlcNAc mutations were verified by Sanger sequencing. *ogt* cDNA (described above) was subcloned into a pMT-3XFLAG-6XHIS vector described in [20], with the epitope at the C-terminus of the ORF. *pCopia-renilla luciferase* and *per*-E-luc constructs were described previously [37]. S2 cells and DES expression medium were obtained from Life technologies (Carlsbad, CA), and transient transfections were performed using Effectene (Qiagen, Valencia, CA) according to manufacturer’s instructions and as previously described [17,20].

### Transcriptional reporter assay in S2 cell culture

Luciferase reporter assays were performed as described [3, 37]. Measurements of luciferase activity were performed using the Dual-Glo luciferase assay system (Promega, Madison, WI) according to the manufacturer’s recommendation on a TriStar LD 941 microplate reader (Berthold Technologies, Oak Ridge, TN).

### Cycloheximide chase experiment in S2 cell culture

pAc-*per*-V5(WT) or mutant variants were transfected into S2 cells with pMT-FLAG-*ogt* or an empty plasmid. 20 hours after transfection, *ogt* expression was induced for 16 hours. Cycloheximide was then added to a final concentration of 10 μg/ml. Cells were harvested and lysed with EB2 [20] at the times indicated. Proteins were analyzed by western blotting as detailed below and in [65].

### Western blotting of protein extracts and protein quantification

S2 cell and adult fly head protein extractions, western blotting, and image analysis, were performed as previously described [58, 65] with modifications. Primary antibodies α-V5 (Invitrogen, Carlsbad, CA) (1:5000) was used to detect CLK-V5 and PER-V5, α-cmyc (9E10, Sigma, St. Louis, MA) (1:5000) to detect PER-CMYC, α-FLAG (Sigma) (1:7000) to detect FLAG-OGT, α-HA 3F10 (Roche, Indianapolis, IN) (1:1000) to detect PER-HA, α-PER (GP5620; RRID:AB_2747405) [57] (1:3000) to detect PER, and α-HSP70 (Sigma) (1:10,000) was used for normalization. Secondary antibodies conjugated with HRP were added at final dilution as follows: α-mouse IgG at 1:5000 for α-V5 detection, 1:7000 for α-FLAG detection, or 1:10,000 for α-HSP70 detection, α-guinea pig IgG at 1:2000 for α-PER detection, and α-rat IgG (1:1000) for α-HA detection of PER-HA. Membranes were imaged and protein levels were quantified using the ChemiDoc MP system with Image Lab software (Bio-Rad). To calculate PER degradation rate, PER intensity was normalized to HSP70 intensity at each time-point, and was then converted to a fraction of the peak value (peak = 1). For quantifying PER levels from fly heads, PER values were normalized against HSP70 intensity at each time-point, and subsequently expressed as a fraction of the peak PER levels.

### Coimmunoprecipitation (co-IP) in S2 cells and in flies

Co-IP assays using protein extracts from S2 cells and fly heads were performed as described [58, 65] with modifications. Proteins were extracted using modified RIPA buffer with the addition of 100μM PUGNAc to preserve O-GlcNAcylation of proteins prior to input analysis by western blotting or Co-IP with appropriate antibodies. Samples were pre-cleared using sepharose beads (Sigma) to reduce nonspecific binding. For co-IP in S2 cells, CLK IP samples were incubated with 15μl α-V5 resin (Sigma) and negative non-specific control IP samples were incubated with 15μl α-HA resin (Sigma). For co-IP in fly heads, CLK IP samples were incubated with 4μl α-CLK antibody (Santa Cruz Biotechnology, Dallas, TX) for 3 hours prior to incubation with 20μl gamma sepharose beads (GE, Pittsburgh, PA) for 1 hour; PER IP samples were incubated with 20μl α-HA resin (Sigma). Immune complexes were resolved by SDS-PAGE as described [58, 65]. IP signal intensity was normalized to intensity of the bait protein. These values were then converted as relative to the peak value of the dataset (peak = 1). Representative data shown are averages of normalized PER or CLK interactions from at least three independent experiments.

### Quantitative RT-PCR to analyze gene expression in fly heads and abdominal fat bodies

Total RNA was extracted from fly heads and abdominal fat bodies using TRI-Reagent (Sigma). cDNA synthesis from total RNA and real-time PCR analysis was performed as previously described [58, 65]. For isolating abdominal fat bodies, flies were collected at the indicated time-points on the first day of DD and immediately transferred in TRI-Reagent (Sigma) for 40 minutes at room temperature with agitation following dissection in RNAlater buffer (Thermo Fischer Scientific). At least 16 flies were dissected for each genotype and time-point. After dissection, fat bodies were rinsed with nuclease-free water twice and resuspended in TRI-Reagent prior to RNA isolation.

### Chromatin immunoprecipitation (ChIP)

ChIP assays were essentially performed as described [58]. qPCR of an intergenic region (FBgn0003638) of the *Drosophila* genome representing background CLK binding was subtracted from input samples. Technical triplicates for the qPCR step were performed for each of the three biological replicates. Two-tailed *t*-tests were used to determine statistical differences between genotypes at each time-point.

### Immunohistochemistry

Adult fly brain immunohistochemistry was performed as described previously [65] with modifications. Briefly, adult flies were entrained in LD cycles for 3 days and collected at the appropriate time-point following incubation with fixative solution (4% paraformaldehyde, 0.2% Triton X-100 in PBS) for at least 40 minutes in the dark at room temperature with gentle rotation. Fixative solution was removed, and then wash solution (0.2% Triton X-100 in PBS) was added to transfer flies into an embryo dish. Brains were dissected using #5 Rubis nano tweezers (Electron Microscopy Sciences, Hatfield, PA). Approximately 10 brains were dissected for each time-point. After dissection, brains were incubated in fixative solution at room temperature for 40 minutes with gentle rotation. Brains were rinsed quickly with wash solution three times prior to three slow washes in wash solution for 10 minutes each with gentle rotation. Wash solution was removed, and blocking solution (0.2% Triton X-100, 5% horse serum in PBS) was added to the brains for 40 minutes at 4°C with gentle rotation. Brains were then incubated with new blocking solution at 4°C overnight with primary antibodies at the following dilutions: α-HA 3F10 antibody (Roche) (1:100), and α-PDF antibody (Developmental Studies Hybridoma Bank, Iowa City, IA) (1:100). After ~18 hr, brains were rinsed quickly with wash solution three times prior to four slow washes with wash solution for 10 minutes each with gentle rotation. Brains were then incubated in secondary antibodies in blocking solution overnight. Secondary antibodies used at the following dilutions were Dylight88-conjugated α-rat (1:100) and Alexa647-conjugated α-mouse (1:100) (Thermo Fischer Scientific). After ~18 hr, brains were rinsed quickly with wash solution three times prior to four slow washes with wash solution for 10 minutes each with gentle rotation. Brains were rinsed quickly with PBS following incubation with 85% glycerol for 15 minutes. Brains were mounted on microscope slides in VectaShield mounting medium (Vector Laboratories, Burlingame, CA) under a #1.5 (17-mm) coverslip. Confocal images were obtained with an Olympus FV1000 Confocal Microscope (Olympus Life Science, Center Valley, PA) and processed with the FV1000 software (Olympus Life Science). Scoring of PER subcellular localization was performed as previously described [66, 67]. At least five brains were used for each genotype and time-point. For statistical analysis, scored LN_v_s from each brain served as one biological replicate. Two-tailed *t*-tests were used to determine statistical differences between genotypes at each time-point.

## Supporting information

S1 Fig Assessing molecular and behavioral rhythms in ^14^N and ^15^N-labeled flies. (A) Eduction graphs showing the average locomotor activity of ^14^N and ^15^N-labeled flies on the indicated days in LD condition or in constant darkness (DD). (B) Double-plot actograms showing average locomotor activity of ^14^N and ^15^N-labeled flies entrained for four days of LD conditions followed by seven days of constant darkness. Tau (τ) represents the period length. N represents sample size. (C) Western blot showing daily cycling of PER in ^14^N and ^15^N-labeled flies.
(TIF)

S2 Fig Workflow in analyzing PER PTMs using *wper*^*0*^; FLAG-*per*(WT) flies. (A) Western blot showing ^15^N PER from fly heads collected at ZT 12 subjected to immunoprecipitation using FLAG resin followed by acetone precipitation. (B) Western blot probing for immunoprecipitated PER from ^14^N fly heads using FLAG antibody at the indicated time-points in LD condition. (C) Coomassie stain showing mixed ^14^N/^15^N PER sample at ZT 12 prior to MS analysis.
(TIF)

S3 Fig Assaying non-O-GlcNAcylatable *per* mutant variants in *Drosophila* S2 cell culture. (A) Western blot showing the expression of different PER variants and CLK in S2 cells for a second replicate of *per*-*luc* assay. HSP70 was used for normalization. (B) Quantification of PER expression in the *per*-*luc* assay from two biological replicates (shown in S3 Fig. and Fig 2B). Asterisk denotes significant differences between PER(WT) and PER(S942A) or PER(S951-T954A) (***P < 0.01). Error bars = SEM from biological replicates. (C) Western blot showing a representative biological replicate of the Cycloheximide (CHX) chase assay in S2 cells coexpressing pAc-*per*(WT)-V5 or pAc-*per*(S942A)-V5 with pMT-FH-*ogt*. HSP70 was used to indicate equal loading and for normalization. (D) Quantification showing rates of PER degradation from the CHX assays in S2 cells. Error bars represent SEM from four independent experiments (n = 4).
(TIF)

S4 Fig Cycling clock gene expression and PER abundance in *per*(S951A/T952/T954A) mutant flies. (A,B) Steady state mRNA expression of *per* and *tim* in heads of *wper*^0^; *p*{*per(WT)-*HA10HIS}, and *wper*^0^; *p*{*per(S951A/T952A/T954A)-*HA10HIS} flies, entrained in 12h:12h LD condition and assayed on LD3 (A) or DD1 (B) (n = 3 biological replicates). Error bars indicate ± SEM (*P-value < 0.05, **P-value < 0.01). (C,D) Western blots and corresponding quantifications comparing PER levels between head extracts of *wper*^0^; *p*{*per(WT)-*HA10HIS} and *wper*^0^; *p*{*per(S951A/T952A/T954A*)-HA10HIS} flies on LD3 (C) or DD1 (D). PER-HA levels were detected using both α-PER (GP5620) (Top) and α-HA (Middle). α-HSP70 was used to indicate equal loading and for normalization (Bottom). Two biological replicates were quantified and depicted in graphical format. Error bars indicate ± SEM (*P-value < 0.05, **P-value < 0.01).
(TIF)

S5 Fig Blocking PER(S942) O-GlcNAcylation does not influence the timing of PER nuclear translocation. (A) Representative confocal images (four sets shown for each genotype per time-point) obtained in the sLN_v_ neurons of adult fly brains collected at the indicated time-points on the third day of LD. PER was visualized using α-HA (3F10) antibodies (stained in green) whereas PDF was visualized by α-PDF antibodies (stained in red). (B) Bar graph showing distribution of nuclear (N), cytoplasmic (C), or both nuclear and cytoplasmic (N+C) of PER in the sLN_v_ neurons at the indicated time-points for *per*(WT) and *per*(S942A) flies (n.s. = not significant). Scoring of PER subcellular localization was performed as previously described [66, 67].
(TIF)

S6 Fig Blocking PER(S942) O-GlcNAcylation promotes PER-CLK interaction and leads to premature CLK removal at *per* and *tim* promoters. (A) Western blot showing a different biological replicate of PER and CLK reciprocal CoIPs from adult fly heads collected at the indicated time-points on LD3. Protein extracts from fly heads were directly analyzed (input) or immunoprecipitated with α-HA (PER) or α-CLK antibodies. Subsequently, immune complexes were subjected to immunoblotting to detect bait or interacting proteins. (B) Western blot showing amount of CLK remaining after CLK IP for ChIP assay at the indicated time-points for *per*(WT) and *per*(S942A) samples to confirm that CLK is not a limiting factor.
(TIF)

S7 Fig Blocking PER(S951/T952/T954) O-GlcNAcylation does not significantly influence PER-CLK interaction. (A) Representative western blots showing CLK and PER reciprocal coIPs in *wper*^0^; *p*{*per(WT)-*HA10HIS} and *wper*^0^; *p*{*per(S951A/T952A/T954A)-*HA10HIS} flies at the indicated time-points. Flies were entrained for 2 days in 12h:12h LD cycles and collected on LD3. Protein extracts from fly heads were directly analyzed (input) or immunoprecipitated with α-HA to detect PER-HA or α-CLK. Immune complexes were then subjected to immunoblotting to detect bait or interacting proteins. (B) Bar graphs showing quantification of reciprocal coIPs to examine PER-CLK binding from two biological replicates. Values for target binding are normalized to amount of bait detected in the IPs. Error bars indicate ± SEM. n.s. = not significant.
(TIF)

S8 Fig Chemoenzymatic O-GlcNAc-labeling approach to detect O-GlcNAc modified PER and OGT in *Drosophila* S2 cells. (A) Western blot showing O-GlcNAc modified and non-O-GlcNAcylated PER-V5 from S2 cell extracts. Protein extracts from S2 cells were directly analyzed by western blotting (input) or subjected to immunoprecipitation using α-V5 resin. Purified PER was chemoenzymatically labeled using a 20-kD PEG mass tag to selectively resolve O-GlcNAc-modified PER in SDS-PAGE. Slower migrating isoforms represent O-GlcNAcylated PER (denoted in green) whereas faster migrating isoforms denote non-O-GlcNAcylated PER. (B) Western blot showing O-GlcNAc-modified OGT-FLAG from S2 cell extracts. Immunoprecipitated OGT was chemoenzymatically labeled using a 10-kD mass tag to selectively resolve O-GlcNAc-modified OGT by SDS-PAGE. Unshifted OGT bands (bottom) represent non-O-GlcNAcylated isoforms of OGT whereas the slower migrating smear represents O-GlcNAc-modified OGT (denoted in green).
(TIF)

S9 Fig CAFE assay to examine daily feeding activity rhythms of flies entrained together with flies used for O-GlcNAc chemoenzymatic labeling experiments shown in Fig 6. Feeding rhythms of *(A)* mixed populations of male and female *wper*^*0*^; *p*{*per(WT)-*HA10HIS} flies (n = 3) or *(B)* male or female flies housed separately over a 24-hour cycle as measured by CAFE assay (n = 3). Error bars indicate ± SEM at individual time-point. Asterisks denote significance difference (*P-value < 0.05, **P-value < 0.01, ***P-value < 0.001) in relative food consumption at the highest feeding period (ZT0-2) compared to other feeding times (ZT8-10, ZT12-14, ZT16-18, and ZT20-22) for *(A)* mixed populations of male and females (black asterisk) or *(B)* separately housed males (grey asterisk) or females (black asterisk). Rhythmicity of feeding activity in females was confirmed by JTK-cycle (*P* < 0.05).
(TIF)

S1 Table Identification of PER phosphorylation sites in fly tissues by label-free mass spectrometry. (DOCX)

S2 Table Mutagenic primer sequences to generate PER O-GlcNAc site mutants. (DOCX)
