# Supplementary figures and images for: O-GlcNAcylation of PERIOD regulates its interaction with CLOCK and timing of circadian transcriptional repression
Source: PLoS Genet. 2019 Jan 31;15(1):e1007953. doi: 10.1371/journal.pgen.1007953 (PMC6372208; doi:10.1371/journal.pgen.1007953)

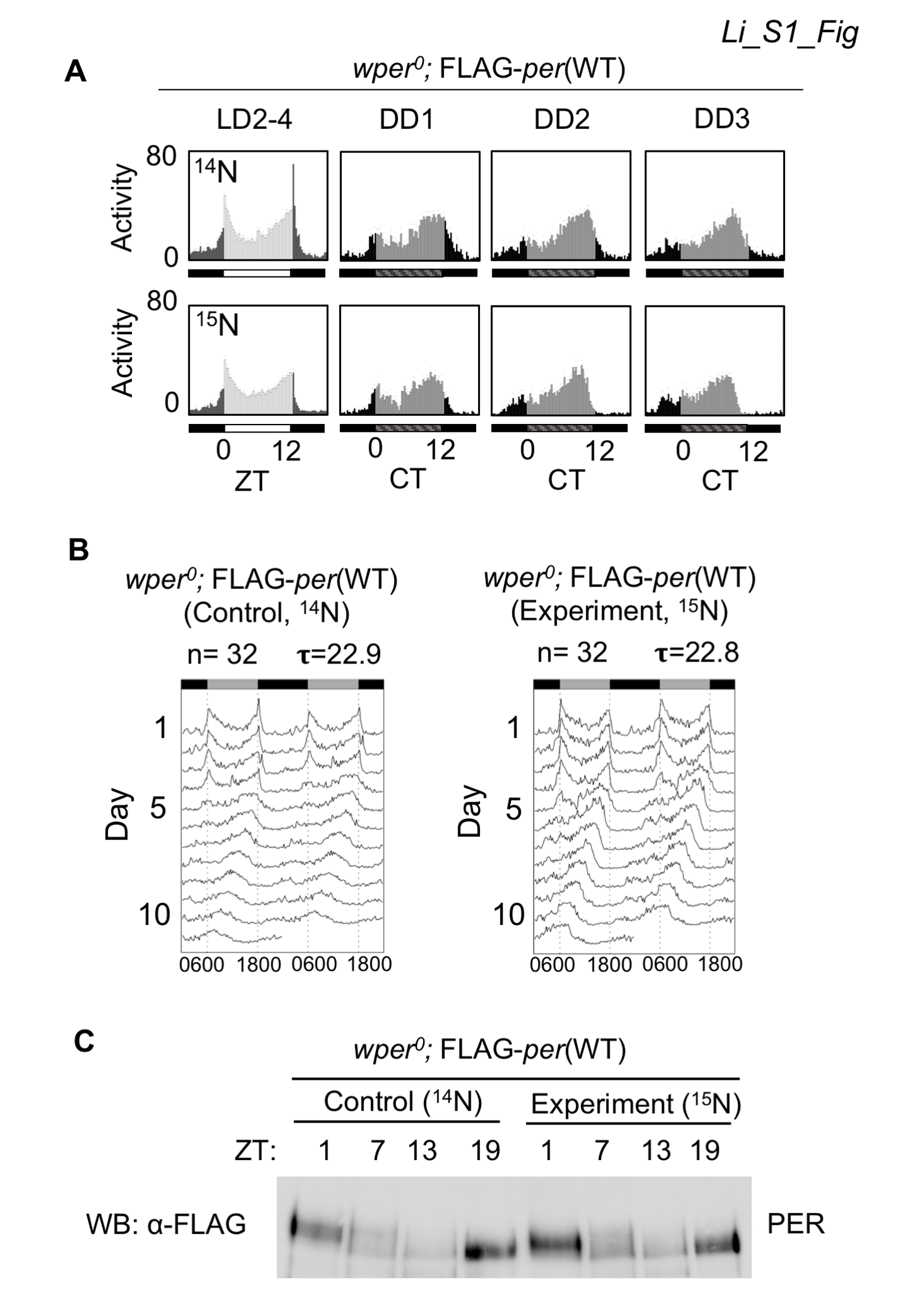

Supplement: S1 Fig — (A) Eduction graphs showing the average locomotor activity of 14N and 15N-labeled flies on the indicated days in LD condition or in constant darkness (DD). (B) Double-plot actograms showing average locomotor activity of 14N and 15N-labeled flies entrained for four days of LD conditions followed by seven days of constant darkness. Tau (τ) represents the period length. N represents sample size. (C) Western blot showing daily cycling of PER in 14N and 15N-labeled flies. (TIF) [file pgen.1007953.s001.tif]

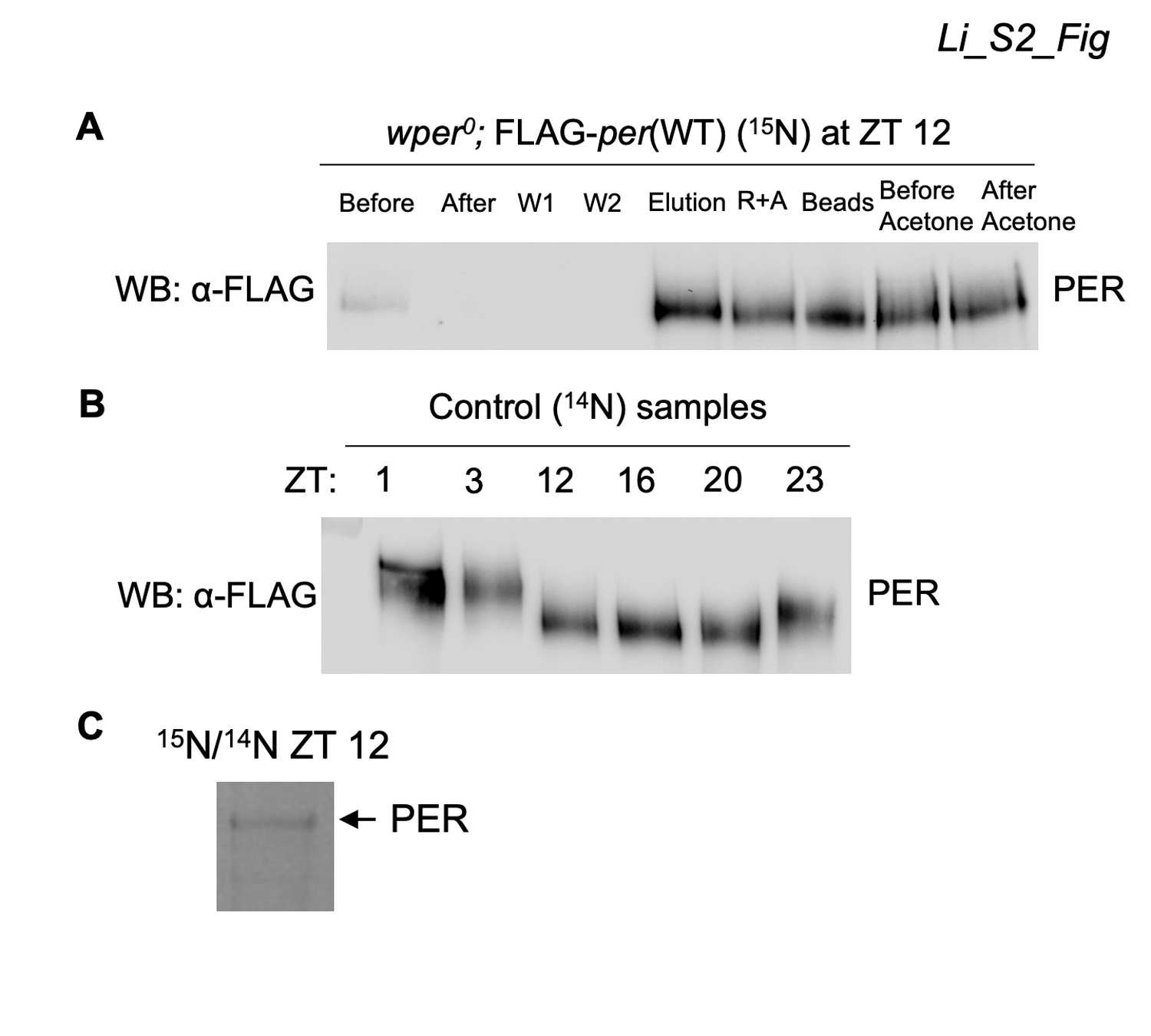

Supplement: S2 Fig — (A) Western blot showing 15N PER from fly heads collected at ZT 12 subjected to immunoprecipitation using FLAG resin followed by acetone precipitation. (B) Western blot probing for immunoprecipitated PER from 14N fly heads using FLAG antibody at the indicated time-points in LD condition. (C) Coomassie stain showing mixed 14N/15N PER sample at ZT 12 prior to MS analysis. (TIF) [file pgen.1007953.s002.tif]

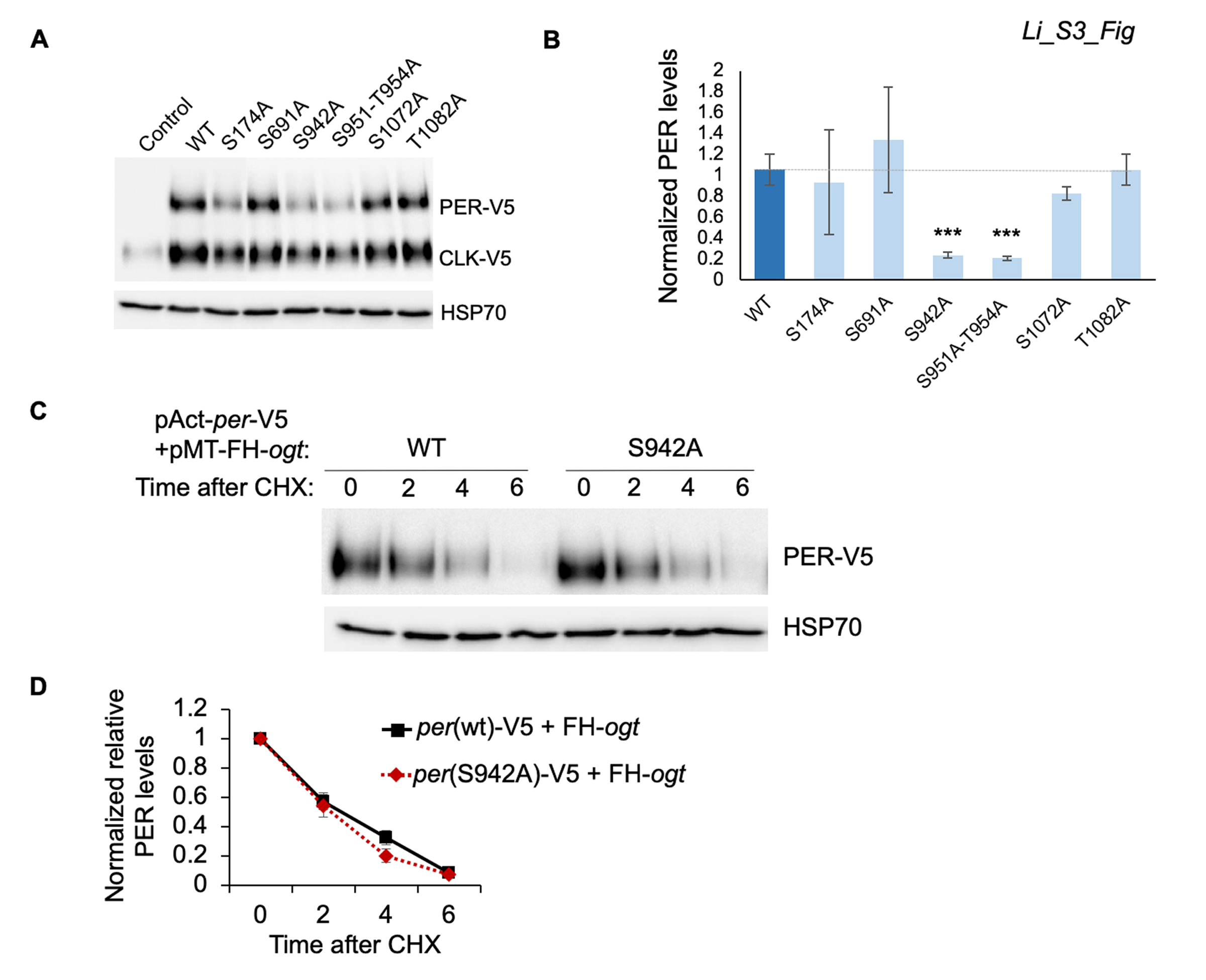

Supplement: S3 Fig — (A) Western blot showing the expression of different PER variants and CLK in S2 cells for a second replicate of per-luc assay. HSP70 was used for normalization. (B) Quantification of PER expression in the per-luc assay from two biological replicates (shown in S3 Fig. and Fig 2B). Asterisk denotes significant differences between PER(WT) and PER(S942A) or PER(S951-T954A) (***P < 0.01). Error bars = SEM from biological replicates. (C) Western blot showing a representative biological replicate of the Cycloheximide (CHX) chase assay in S2 cells coexpressing pAc-per(WT)-V5 or pAc-per(S942A)-V5 with pMT-FH-ogt. HSP70 was used to indicate equal loading and for normalization. (D) Quantification showing rates of PER degradation from the CHX assays in S2 cells. Error bars represent SEM from four independent experiments (n = 4). (TIF) [file pgen.1007953.s003.tif]

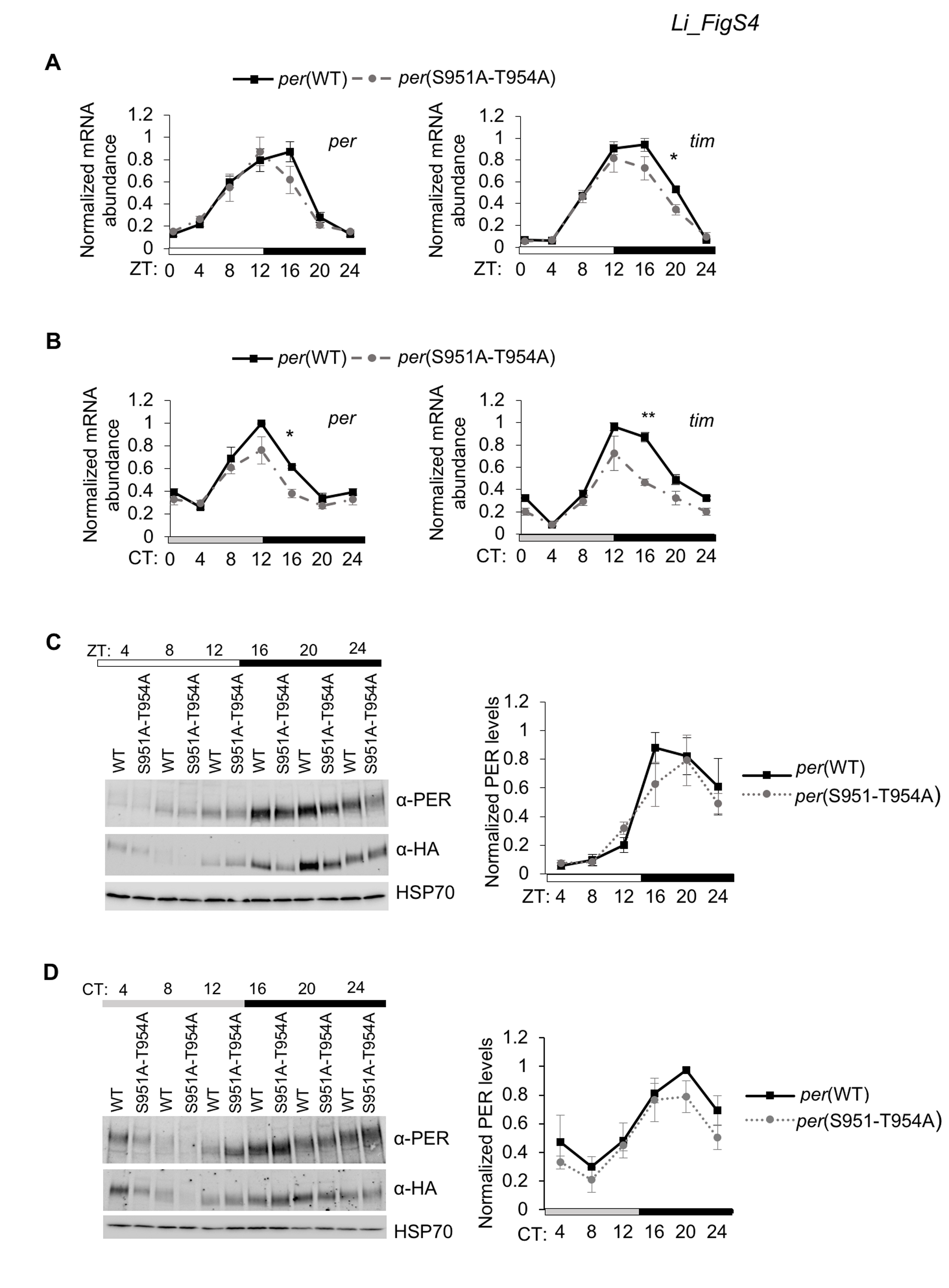

Supplement: S4 Fig — (A,B) Steady state mRNA expression of per and tim in heads of wper0; p{per(WT)-HA10HIS}, and wper0; p{per(S951A/T952A/T954A)-HA10HIS} flies, entrained in 12h:12h LD condition and assayed on LD3 (A) or DD1 (B) (n = 3 biological replicates). Error bars indicate ± SEM (*P-value < 0.05, **P-value < 0.01). (C,D) Western blots and corresponding quantifications comparing PER levels between head extracts of wper0; p{per(WT)-HA10HIS} and wper0; p{per(S951A/T952A/T954A)-HA10HIS} flies on LD3 (C) or DD1 (D). PER-HA levels were detected using both α-PER (GP5620) (Top) and α-HA (Middle). α-HSP70 was used to indicate equal loading and for normalization (Bottom). Two biological replicates were quantified and depicted in graphical format. Error bars indicate ± SEM (*P-value < 0.05, **P-value < 0.01). (TIF) [file pgen.1007953.s004.tif]

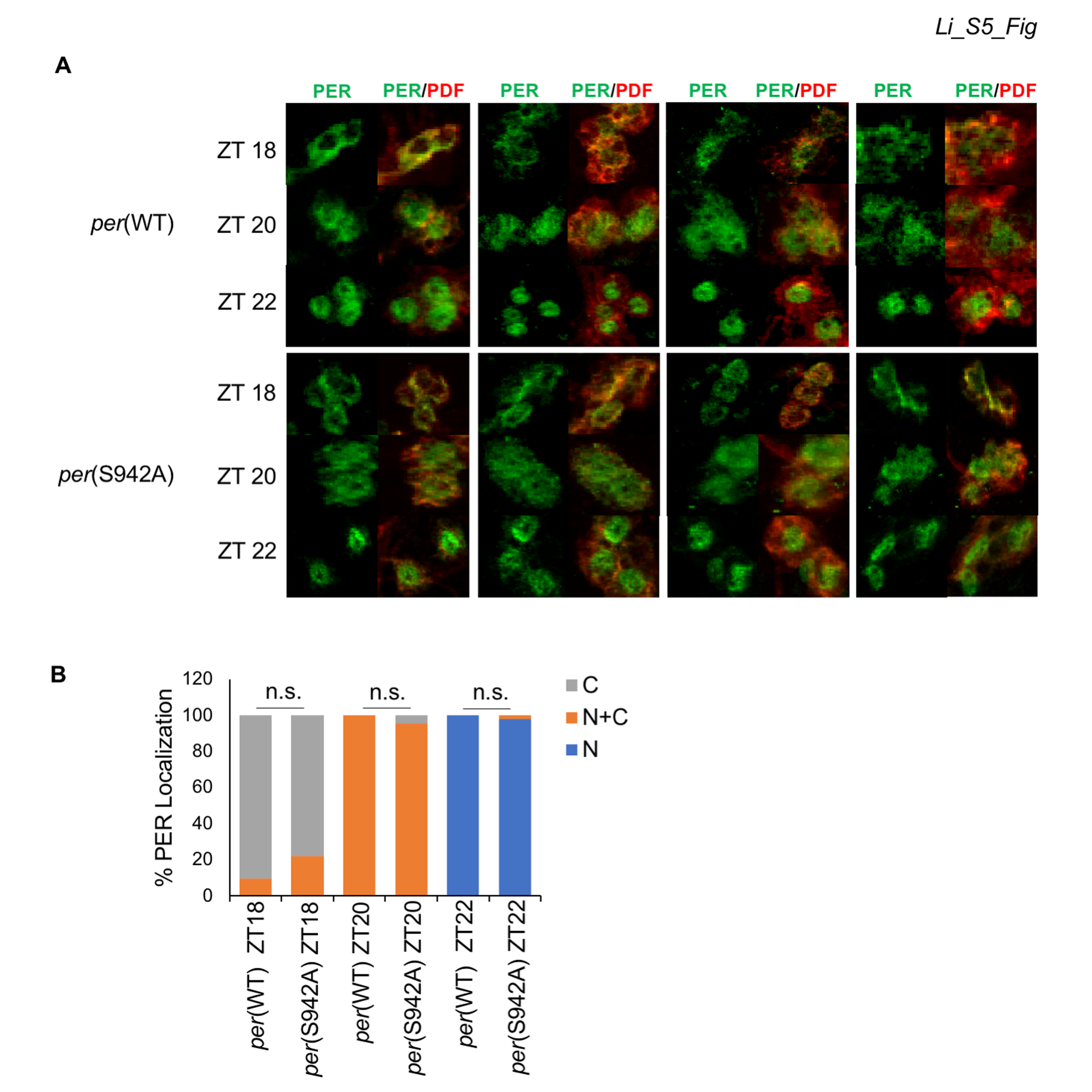

Supplement: S5 Fig — (A) Representative confocal images (four sets shown for each genotype per time-point) obtained in the sLNv neurons of adult fly brains collected at the indicated time-points on the third day of LD. PER was visualized using α-HA (3F10) antibodies (stained in green) whereas PDF was visualized by α-PDF antibodies (stained in red). (B) Bar graph showing distribution of nuclear (N), cytoplasmic (C), or both nuclear and cytoplasmic (N+C) of PER in the sLNv neurons at the indicated time-points for per(WT) and per(S942A) flies (n.s. = not significant). Scoring of PER subcellular localization was performed as previously described [66, 67]. (TIF) [file pgen.1007953.s005.tif]

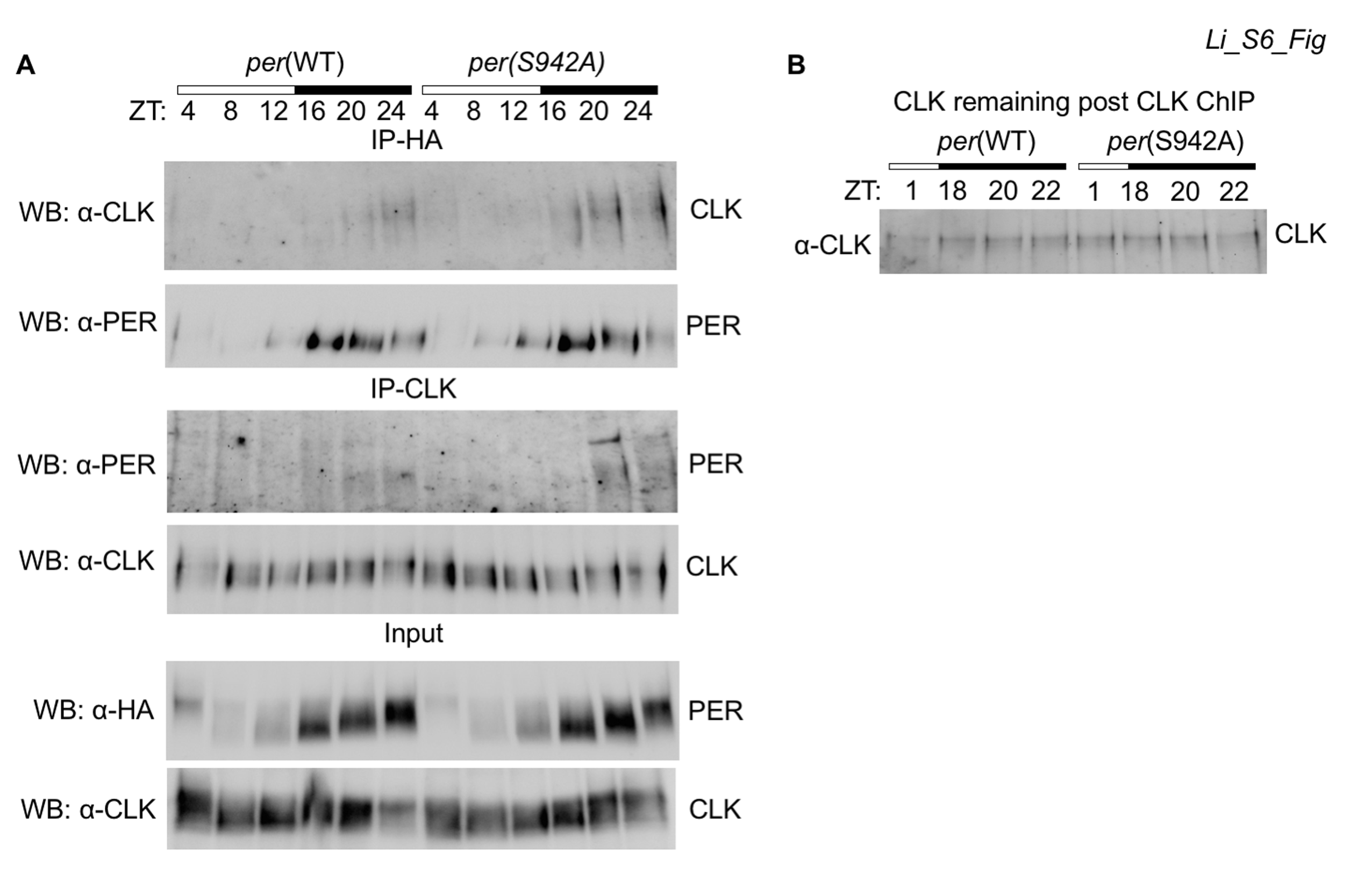

Supplement: S6 Fig — (A) Western blot showing a different biological replicate of PER and CLK reciprocal CoIPs from adult fly heads collected at the indicated time-points on LD3. Protein extracts from fly heads were directly analyzed (input) or immunoprecipitated with α-HA (PER) or α-CLK antibodies. Subsequently, immune complexes were subjected to immunoblotting to detect bait or interacting proteins. (B) Western blot showing amount of CLK remaining after CLK IP for ChIP assay at the indicated time-points for per(WT) and per(S942A) samples to confirm that CLK is not a limiting factor. (TIF) [file pgen.1007953.s006.tif]

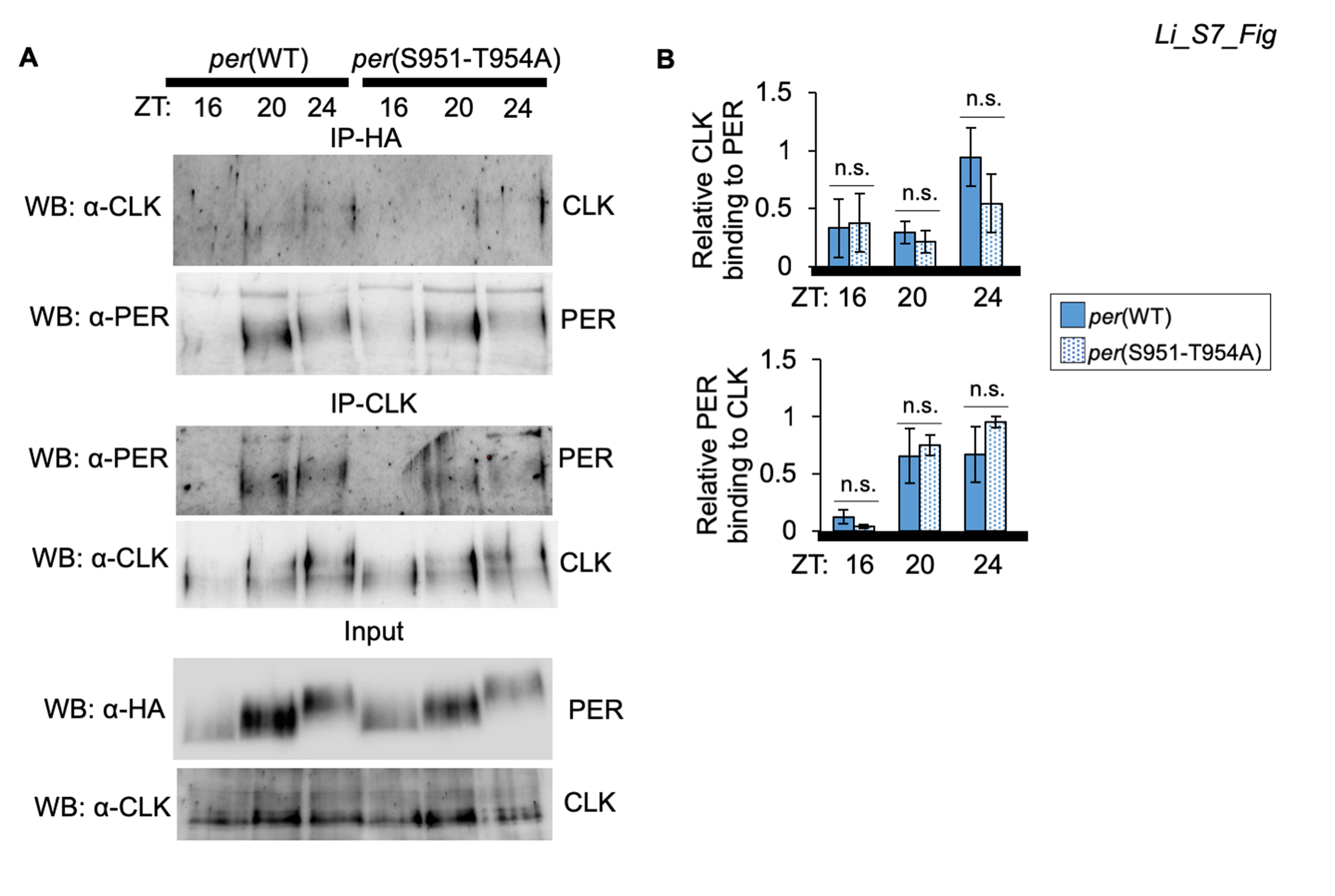

Supplement: S7 Fig — (A) Representative western blots showing CLK and PER reciprocal coIPs in wper0; p{per(WT)-HA10HIS} and wper0; p{per(S951A/T952A/T954A)-HA10HIS} flies at the indicated time-points. Flies were entrained for 2 days in 12h:12h LD cycles and collected on LD3. Protein extracts from fly heads were directly analyzed (input) or immunoprecipitated with α-HA to detect PER-HA or α-CLK. Immune complexes were then subjected to immunoblotting to detect bait or interacting proteins. (B) Bar graphs showing quantification of reciprocal coIPs to examine PER-CLK binding from two biological replicates. Values for target binding are normalized to amount of bait detected in the IPs. Error bars indicate ± SEM. n.s. = not significant. (TIF) [file pgen.1007953.s007.tif]

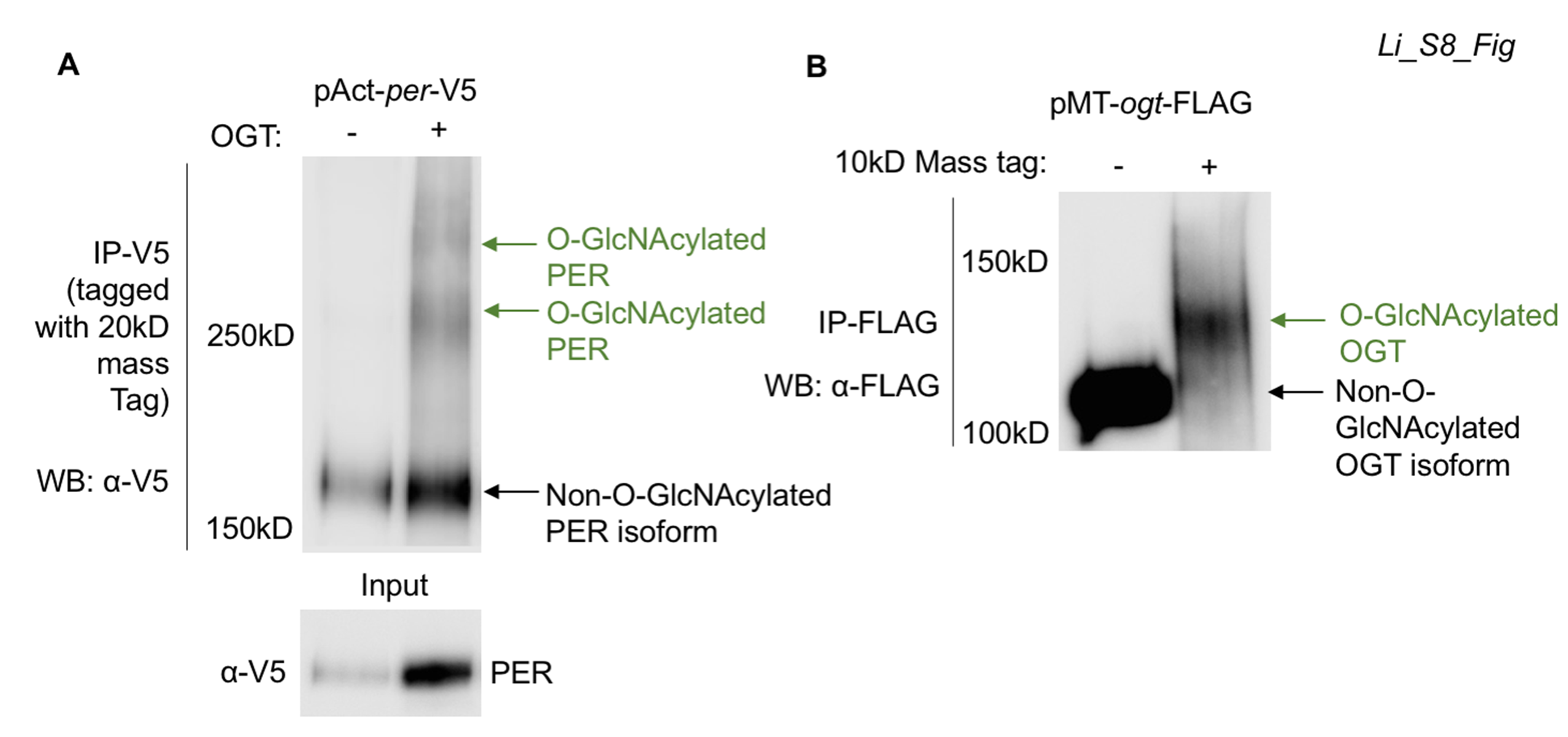

Supplement: S8 Fig — (A) Western blot showing O-GlcNAc modified and non-O-GlcNAcylated PER-V5 from S2 cell extracts. Protein extracts from S2 cells were directly analyzed by western blotting (input) or subjected to immunoprecipitation using α-V5 resin. Purified PER was chemoenzymatically labeled using a 20-kD PEG mass tag to selectively resolve O-GlcNAc-modified PER in SDS-PAGE. Slower migrating isoforms represent O-GlcNAcylated PER (denoted in green) whereas faster migrating isoforms denote non-O-GlcNAcylated PER. (B) Western blot showing O-GlcNAc-modified OGT-FLAG from S2 cell extracts. Immunoprecipitated OGT was chemoenzymatically labeled using a 10-kD mass tag to selectively resolve O-GlcNAc-modified OGT by SDS-PAGE. Unshifted OGT bands (bottom) represent non-O-GlcNAcylated isoforms of OGT whereas the slower migrating smear represents O-GlcNAc-modified OGT (denoted in green). (TIF) [file pgen.1007953.s008.tif]

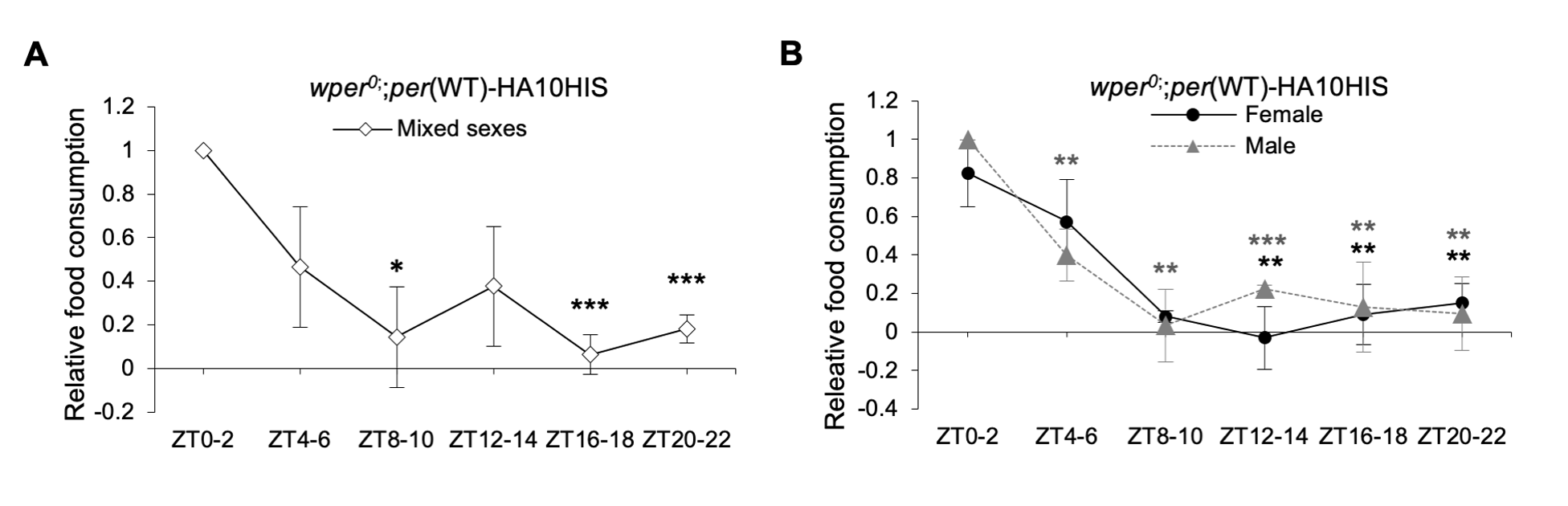

Supplement: S9 Fig — Feeding rhythms of (A) mixed populations of male and female wper0; p{per(WT)-HA10HIS} flies (n = 3) or (B) male or female flies housed separately over a 24-hour cycle as measured by CAFE assay (n = 3). Error bars indicate ± SEM at individual time-point. Asterisks denote significance difference (*P-value < 0.05, **P-value < 0.01, ***P-value < 0.001) in relative food consumption at the highest feeding period (ZT0-2) compared to other feeding times (ZT8-10, ZT12-14, ZT16-18, and ZT20-22) for (A) mixed populations of male and females (black asterisk) or (B) separately housed males (grey asterisk) or females (black asterisk). Rhythmicity of feeding activity in females was confirmed by JTK-cycle (P < 0.05). (TIF) [file pgen.1007953.s009.tif]
