# Supplementary material for: O-GlcNAcylation of PERIOD regulates its interaction with CLOCK and timing of circadian transcriptional repression
Source: PLoS Genet. 2019 Jan 31;15(1):e1007953. doi: 10.1371/journal.pgen.1007953 (PMC6372208; doi:10.1371/journal.pgen.1007953)
Supplement: S1 Table — (DOCX) [file pgen.1007953.s010.docx]

**S1 Table. Identification of PER phosphorylation sites in fly tissues by label-free mass spectrometry**

| Modified Residue^a^ | Peptide sequence^b^ |  |  |
| --- | --- | --- | --- |
|  |  | Present in ^14^N/^15^N MS dataset?^c^ | Previously identified in S2 cell culture?^d^ |
| S-40 | LSGSHSSGSSGYGGKPSTQASSSDMIIKR | No | Yes |
| S-47 or S-48 | LSGSHSSGSSGYGGKPSTQASSSDMIIKR | Yes | Yes |
| S-48 | LSGSHSSGSSGYGGKPSTQASSSDMIIKR | Yes | Yes |
| [S-149; S-151; S-153] | ELQDQQHGEDHSEPQAIEQLQQEEEEDQSGSESEADRVEGVAK | Yes | Yes^,,^ |
| S-149 | ELQDQQHGEDHSEPQAIEQLQQEEEEDQSGSESEADRVEGVAK | Yes | Yes^♐,,^ |
| S-174 | SEAAQSFPIPSPLSVTIVPPSMGGcGGVGHAAGLDSGLAK | Yes | Yes^,^ |
| [S-174; S-177] | SEAAQSFPIPSPLSVTIVPPSMGGcGGVGHAAGLDSGLAK | No | Yes |
| T-179 | SEAAQSFPIPSPLSVTIVPPSMGGcGGVGHAAGLDSGLAK | No | No |
| S-596 | DSVMLGEISPHHDYYDSK | Yes | Yes^,,^ |
| Y-601 | DSVMLGEISPHHDYYDSKSSTETPPSYNQLNYNENLLR | No | No |
| [S-596; Y-601] | DSVMLGEISPHHDYYDSKSSTETPPSYNQLNYNENLLR | No | No |
| T-608 | SSTETPPSYNQLNYNENLLR | Yes | Yes |
| T-610 | SSTETPPSYNQLNYNENLLR | Yes | Yes^,,,^ |
| [S-826; S-828] | RGGSHSWEGEANKPK | Yes | Yes^,,^ |
| S-828 | GGSHSWEGEANKPK | Yes | Yes^,^ |
| T-853 | GAAGSAGGAVGTGGVGSGGAGVAGGGGSGTGVAGTPEGR | Yes | No |
| S-865 | GAAGSAGGAVGTGGVGSGGAGVAGGGGSGTGVAGTPEGR | No | Yes |
| S-876 | GAAGSAGGAVGTGGVGSGGAGVAGGGGSGTGVAGTPEGR | Yes | Yes^,^ |
| T-878 | GAAGSAGGAVGTGGVGSGGAGVAGGGGSGTGVAGTPEGR | No | No |
| [S-1102; S-1103] | KVPGAFHSVTTPAQVQRPSSQSASVK | Yes | Yes^,^ |
| S-1107 | KVPGAFHSVTTPAQVQRPSSQSASVK | No | No |
| S-1119 | TEPGSSAAVSDPCKK | Yes | No |
| [S-1129; S-1130] | EVPDSSPIPSVMGDYNSDPPCSSSNPANNKK | Yes | Yes^,^ |
| S-1130 | EVPDSSPIPSVMGDYNSDPPCSSSNPANNKK | Yes | Yes^,^ |
| S-1141 | EVPDSSPIPSVMGDYNSDPPCSSSNPANNKK | Yes | No |
| S-1163 | YTDSNGNSDDMDGSSFSSFYSSFIK | No | No |
| [S-1185; S-1187] | TTDGSESPPDTEKDPK | Yes | Yes^,^ |
| S-1187 | TTDGSESPPDTEKDPK | Yes | Yes^,^ |

^a^Residues within brackets indicate potential phosphorylated residue present within each peptide.

^b^Potential modified residues are underlined.

^a^See Table 1.

^d^Comparison of PER phosphorylation sites identified in this study with those previously identified from mass spectrometry analysis in *Drosophila* S2 cells as reported in Chiu et al. 2008 [17], Kivimae et al. 2008 [18], Ko et al. 2010 [19], and Garbe et al. 2013 [21].
