# Supplementary material for: O-GlcNAcylation of PERIOD regulates its interaction with CLOCK and timing of circadian transcriptional repression
Source: PLoS Genet. 2019 Jan 31;15(1):e1007953. doi: 10.1371/journal.pgen.1007953 (PMC6372208; doi:10.1371/journal.pgen.1007953)
Supplement: S2 Table — (DOCX) [file pgen.1007953.s011.docx]

**S2 Table. Mutagenic primer sequences to generate PER O-GlcNAc site mutants**

| O-GlcNAc site | Primer | Sequence (5´-3´) |
| --- | --- | --- |
| Ser^174^ | Forward  Reverse | AGCTTTCCGATTCCCGCGCCGCTATCCGTCACC  GGTGACGGATAGCGGCGCGGGAATCGGAAAGCT |
| Ser^691^ | Forward  Reverse | AGTGTGACAAATACGGCCATTGCCGGCACTGGTGGC  GCCACCAGTGCCGGCAATGGCCGTATTTGTCACACT |
| Ser^942^ | Forward  Reverse | GTGGGCATCACACCGGCCGTTCACTCCACGCAC  GTGCGTGGAGTGAACGGCCGGTGTGATGCCCAC |
| Ser^951^-Thr^952^-Thr^954^ | Forward  Reverse | ACCGCCCGTTCACGCCGCGCACGCGGCCATGGCCCAGAGC  GCTCTGGGCCATGGCCGCGTGCGCGGCGTGAACGGGCGGT |
| Ser^1072^ | Forward  Reverse | TCCGCGTAGAACAAGGCGGTGTAGACGACCACG  CGTGGTCGTGTACACCGCCTTGTTGTACGCGGA |
| Ser^1080^ | Forward  Reverse | ACGACCACGCCGGCGGCCATGACGAAGAAGGTG  CACCTTCTTCGTCATGGCCGCCGGCGTGGTCGT |
| Thr^1082^ | Forward  Reverse | ACGCCGGCGTCCATGGCGAAGAAGGTGCCGGGTG  CACCCGGCACCTTCTTCGCCATGGACGCCGGCGT |
